# First Generation of Antioxidant Precursors for Bioisosteric Se-NSAIDs: Design, Synthesis, and In Vitro and In Vivo Anticancer Evaluation

**DOI:** 10.3390/antiox12091666

**Published:** 2023-08-24

**Authors:** Sandra Ramos-Inza, Cesar Aliaga, Ignacio Encío, Asif Raza, Arun K. Sharma, Carlos Aydillo, Nuria Martínez-Sáez, Carmen Sanmartín, Daniel Plano

**Affiliations:** 1 Department of Pharmaceutical Technology and Chemistry, University of Navarra, Irunlarrea 1, 31008 Pamplona, Spain; sramos.2@alumni.unav.es (S.R.-I.); caydillo@unav.es (C.A.); nmartinezsa@unav.es (N.M.-S.); 2 Instituto de Investigación Sanitaria de Navarra (IdiSNA), Irunlarrea 3, 31008 Pamplona, Spain; 3 Department of Pharmacology, Penn State Cancer Institute, CH72, Penn State College of Medicine, 500 University Drive, Hershey, PA 17033, USA; caliaga@pennstatehealth.psu.edu (C.A.); mraza@pennstatehealth.psu.edu (A.R.); 4 Department of Health Sciences, Public University of Navarra, Avda. Barañain s/n, 31008 Pamplona, Spain; ignacio.encio@unavarra.es

**Keywords:** selenium, diacyl diselenide, NSAID, antiproliferative agents, antioxidant, colon cancer, mouse xenograft model

## Abstract

The introduction of selenium (Se) into organic scaffolds has been demonstrated to be a promising framework in the field of medicinal chemistry. A novel design of nonsteroidal anti-inflammatory drug (NSAID) derivatives based on a bioisosteric replacement via the incorporation of Se as diacyl diselenide is reported. The antioxidant activity was assessed using the DPPH radical scavenging assay. The new Se-NSAID derivatives bearing this unique combination showed antioxidant activity in a time- and dose-dependent manner, and also displayed different antiproliferative profiles in a panel of eight cancer cell lines as determined by the MTT assay. Ibuprofen derivative **5** was not only the most antioxidant agent, but also selectively induced toxicity in all the cancer cell lines tested (IC_50_ < 10 µM) while sparing nonmalignant cells, and induced apoptosis partially without enhancing the caspase 3/7 activity. Furthermore, NSAID derivative **5** significantly suppressed tumor growth in a subcutaneous colon cancer xenograft mouse model (10 mg/kg, TGI = 72%, and T/C = 38%) without exhibiting any apparent toxicity. To our knowledge, this work constitutes the first report on in vitro and in vivo anticancer activity of an unprecedented Se-NSAID hybrid derivative and its rational use for developing precursors for bioisosteric selenocompounds with appealing therapeutic applications.

## 1. Introduction

Selenium (Se) is an essential trace element that has been recognized to play an important role in human health [1]. Se exerts its biological activities mainly in the form of selenocysteine, a proteogenic amino acid bearing the selenol functionality (SeH), which is part of selenoproteins [2]. The unique features of this selenol moiety enable selenoproteins to accomplish a wide variety of different physiological functions [2], including protection against oxidative stress, immune response, cellular differentiation [3], and maintenance of the redox homeostasis [4], among others. Selenoprotein-mediated mechanisms are also involved in the prevention, onset, and clinical outcome of various diseases such as cancer [2]. Moreover, a positive relationship between Se status and a favorable prognosis of these diseases has been generally observed [2].

In this context, a large body of evidence supports the role of Se in preventing cancer incidence and blocking tumor metastasis [5]. Recently, a potential benefit of the dietary intake of certain microelements, including Se, on colorectal cancer was also reported [6]. Furthermore, epidemiological studies have shown that low Se intake and low plasma Se levels could lead to an increased incidence of several cancers [7,8,9]. Moreover, Se supplementation alongside traditional therapies has been shown to increase the efficacy of anticancer drugs, limit associated side outcomes, and improve the general conditions of the patients [10,11,12].

Accordingly, over recent decades Se-containing compounds have attracted growing interest as anticancer agents due to their high efficacy and selectivity against cancer cells [4]. In this context, several Se-containing compounds have been proven to exert potent anticancer activity in several xenograft models [13,14,15], and thus they have been considered appealing agents in the field of drug discovery for cancer therapy [1,4,16,17]. The proposed mechanisms involved in the anticancer activity of Se-containing compounds include involvement in angiogenesis, induction of cell cycle arrest, and the initiation of cell death triggered by diverse processes such as apoptosis, necrosis, autophagy, ferroptosis, necroptosis, or entosis, among others [16].

The efficacy of selenocompounds as anticancer agents is correlated with their metabolic route, dose, and the chemical form of Se [5,18]. Hence, the main Se entities that have been widely explored to date are selenides [19,20,21,22], selenocyanates [22,23,24,25,26], isoselenocyanates [27,28,29], selenazo heterocyclic compounds [13,30,31], selenoesters [32,33,34], selenoureas [35,36,37,38,39], and diselenides [19,22,23,24,25,39,40]. Additionally, several compounds bearing these functionalities have also been reported to have a potent radical scavenging capacity, allowing the design of molecules with an interesting dual profile as antioxidant and antiproliferative agents [19,25,37,38].

Furthermore, among the scaffolds that have attracted great attention due to their therapeutic properties, nonsteroidal anti-inflammatory drugs (NSAIDs) have recently emerged as promising frameworks to be modified through the incorporation of different Se functionalities [41]. NSAIDs are prominent agents with applications in cancer treatment and prevention [42] aside from their traditional analgesic, antipyretic, and anti-inflammatory prescriptions. Numerous meta-analyses and systematic reviews to date have supported their chemopreventive and chemotherapeutic potential [43,44,45,46], especially in colon cancer [47,48,49,50,51]. Hence, the use of aspirin (ASA) and ibuprofen (Ibup) for reducing the high risk of colorectal cancer with DNA mismatch repair (MMR) genes is reported [52]. In another study, a synergistic effect of sulindac and bexarotene was observed in familial adenomatous polyposis (FAP) patients [53]. Likewise, another recent cohort study suggested an association between NSAID exposure and reduced cancer recurrence without increasing the risk of anastomotic leakage [54]. The key mechanisms for the therapeutic activity of NSAIDs are the inhibition of cyclooxygenases (COX) and suppression of the production of prostaglandins (PGs) [55]. Interestingly, human colon cancers have elevated PGE_2_ levels, and overexpression of COX has been found in 45% of colon adenomas and 85% of colon carcinomas [56]. Additionally, several studies have shown that the anticancer activity of NSAIDs could also be exerted through COX-independent mechanisms [57], such as the regulation of the Wnt pathway [58] or the DLL1 and NOTCH1 expression [59].

**Figure 1:**
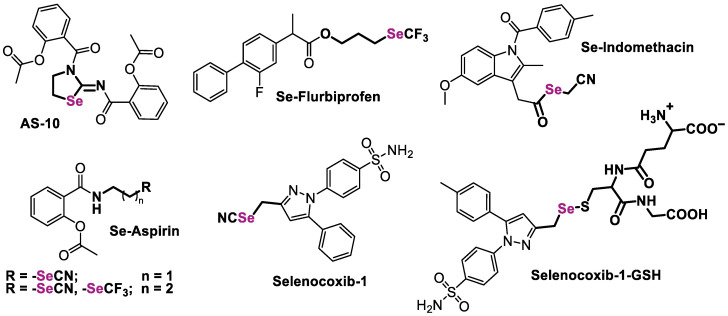
Chemical structures of some NSAID derivatives bearing Se [60,61,62,63,64,65].

In this context, the development of new NSAID analogs based on the combination of NSAIDs and Se entities has attracted great attention (Figure 1). The ASA analogs bearing Se through a selenazolidine heterocycle (AS-10) [60] or a selenocyanate group (Se-Aspirin) [61] showed potent growth inhibition via different mechanisms in pancreatic and colorectal cancer cells. Other Se-NSAID derivatives with a longer carbon chain elongation or even with trifluoromethyl selenides attached through an amide [62] or ester [63] group also displayed potent antiproliferative activity via induction of apoptosis. Remarkably, a derivative of celecoxib synthesized through the modification of the position-3 of the pyrazole ring with methylene selenocyanate, namely selenocoxib-1, showed better efficacy against prostate cancer cells than celecoxib by inducing apoptosis and decreasing HIF-1α, p-AKT, and Bcl-2 levels [64]. Likewise, the glutathione conjugate (selenocoxib-1-GSH) showed potent tumor growth inhibition associated with the suppression of COX-2 and PI3K/AKT signaling pathways [65]. Furthermore, we recently reported a library of Se-NSAID derivatives functionalized with a selenoester group. Among them, Se-indomethacin was identified as a potent antiproliferative agent and inducer of apoptosis in breast cancer cells [66].

Therefore, considering the chemotherapeutic activity of NSAIDs, the literature supporting the structural modification of NSAID scaffolds to create more potent anticancer drugs, and the reported antitumor profile of organoselenium compounds as chemopreventive and chemotherapeutic agents in preclinical models, we hypothesized that the combination of NSAIDs and a diacyl diselenide group could lead to novel Se-NSAID analogs with a therapeutic application as precursors of active molecules. To our knowledge, this is the first study that reports the therapeutic potential of precursors for bioisosteric Se-NSAIDs based on the diacyl diselenide moiety, and specifically their anticancer effect both in vitro and in vivo, along with their preliminary evaluation as antioxidant agents.

Herein, we designed and synthesized five new Se-NSAID hybrid analogs as precursors for the corresponding selenols, which isosterically replace the hydroxide (-OH) of the carboxylic acid functionality of the NSAIDs by -SeH, and evaluated their antiproliferative activity in several cancer cell lines. The selectivity of the new compounds was determined in two nonmalignant cell lines. The following five NSAIDs were selected to be structurally modified: aspirin (ASA), indomethacin (Ind), naproxen (Nap), ketoprofen (Ket), and ibuprofen (Ibup). Compared with our previously reported methylselenoester NSAIDs [66], Ibup was added to this set of newly synthesized diacyldiselenides. The capacity of the new diacyl diselenides to scavenge free radicals was also studied in vitro at different time points and concentrations. The most active and selective compound **5** was further evaluated to assess its capability to induce apoptosis and inhibit tumor growth in a subcutaneous colon cancer xenograft mouse model in order to determine if the novel combination of Se and NSAID scaffolds through a diacyl diselenide group is an appealing new framework for therapeutic development.

## 2. Materials and Methods

### 2.1. Chemistry

#### 2.1.1. General Remarks

Starting materials, reagents, and solvents were purchased from commercial suppliers and used as received without further purification. Reaction courses were monitored by thin-layer chromatography (TLC) performed on precoated silica gel 60 F254 aluminum sheets (Merck, Darmstadt, Germany), and the spots were visualized under UV light. The crude reaction products were purified by silica gel column chromatography using silica gel 60 Å (0.040–0.063 mm, Merck, Darmstadt, Germany) or by flash chromatography, using hexane/ethyl acetate as the elution solvent. ^1^H, ^13^C, and ^77^Se nuclear magnetic resonance (NMR) spectra were recorded on a Bruker Avance Neo 400 MHz operating at 400, 100, and 76 MHz, respectively, using CDCl_3_ as solvent and TMS as the internal standard. Chemical shifts are reported in δ values (ppm) and coupling constants (*J*) values are reported in hertz (Hz). Melting points (mp) were determined with a Mettler FP82 + FP80 apparatus (Greifensee, Switzerland). High-resolution mass spectrometry (HRMS) was performed on a Micromass Q-TOF mass spectrometer. The mass spectrum of compound **5** was obtained by the direct injection of a methanolic solution of **5** in an Expression^S^ CMS Advion mass spectrometer with a MX-Class pump operating in a negative ionization mode. For purity testing, quantitative NMR (qNMR) spectroscopy was performed in concordance with a standardized protocol [67]. Dimethyl sulfone (TraceCERT certified reference compound, lot no. BCBQ8884V, purity: 99.96%) purchased from Sigma-Aldrich (Alcobendas, Madrid, Spain) was used as an internal calibrant. All the newly synthesized diacyl diselenides were >95% pure.

#### 2.1.2. General Procedure for the Preparation of the Diacyl Diselenide Derivatives

The NSAID acyl chloride (2 mmol) was added to a water solution of sodium hydrogen selenide (2 mmol) formed in situ by the reaction of powdered selenium (2 mmol) with sodium borohydride (4 mmol) in water, following the procedure previously reported by Sanmartin et al. [68] with modifications. THF (5 mL) was added, and the mixture was stirred at room temperature for 30–60 min. The reaction mixture was then extracted with methylene chloride (3 × 20 mL). The organic layers were dried with sodium sulfate and concentrated in vacuo. The resulting product was purified using silica gel chromatography or flash chromatography with a hexane/ethyl acetate gradient. For NSAIDs Ind, Ket, and Ibup, the synthesis of the acyl chloride derivatives was necessary prior to the formation of the diacyl diselenide compounds. These derivatives were obtained by the reaction of the carboxylic acid reagent (1 mmol) with oxalyl chloride (3 mmol) in methylene chloride (25 mL). In the case of the reactions involving Ket or Ibup acids, *N*,*N*-dimethylformamide (0.1 mL) was also added. The mixture was stirred overnight at room temperature. The crude product was isolated by the evaporation of the solvent in vacuo and washed with methylene chloride (2 × 15 mL). The acyl chlorides synthesized were then used without further purification.

*2-Acetoxybenzoic diselenoperoxyanhydride* (**1**). The title compound was synthesized from *O*-acetylsalicyloyl chloride, selenium, and sodium borohydride according to the general procedure described above. A yellow solid was obtained. Yield: 43%; mp: 55–58 °C. ^1^H NMR (400 MHz, CDCl_3_) δ 2.40 (s, 6H, CH_3_, CH_3′_); 7.21 (dd, 2H, *J* = 8.2 and 0.9 Hz, H_3_, H_3′_); 7.39 (td, 2H, *J* = 7.7 and 1.1 Hz, H_5_, H_5′_); 7.63 (td, 2H, *J* = 8.1, 7.6 and 1.6 Hz, H_4_, H_4′_); 8.02 (dd, 2H, *J* = 7.8 and 1.6 Hz, H_6_, H_6′_). ^13^C NMR (100 MHz, CDCl_3_) δ 21.5 (CH_3_, CH_3′_), 124.3, 126.6, 129.8, 130.4, 134.6, 148.0 (C_aryl_), 169.1 (CH_3_-C=O), 184.5 (Se-C=O). ^77^Se NMR (76 MHz, CDCl_3_) δ 659. HRMS *m/z*: calcd for C_18_H_14_O_6_Se_2_ [M + Na]^+^, 508.9013; found, 508.9025. Purity (qNMR: 25 °C, CDCl_3_, m_s_ = 1.8 mg, m_IC_ = 4.0 mg): 97.1%.

*2-(1-(4-Chlorobenzoyl)-5-methoxy-2-methyl-1H-indol-3-yl)acetic diselenoperoxyanhydride* (**2**). The title compound was synthesized from 1-(4-chlorobenzoyl)-5-methoxy-2-methyl-3-indoleacetic acid, oxalyl chloride, selenium, and sodium borohydride according to the general procedure described above. A yellow solid was obtained. Yield: 13%; mp: 78–79 °C. ^1^H NMR (400 MHz, CDCl_3_) δ 2.44 (s, 6H, CH_3_, CH_3′_); 3.84 (s, 6H, OCH_3_, OCH_3′_); 4.00 (s, 4H, CH_2_, CH_2′_); 6.71 (dd, 2H, *J* = 9.0 and 2.4 Hz, H_6A_, H_6′A_); 6.91 (d, 2H, *J* = 2.2 Hz, H_4A_, H_4′A_); 6.93 (d, 2H, *J* = 9.1 Hz, H_7A_, H_7′A_); 7.48 (dd, 4H, *J* = 8.4 Hz, H_3B_ + H_5B_, H_3′B_ + H_5′B_); 7.68 (dd, 4H, *J* = 8.4 Hz, H_2B_ + H_6B_, H_2′B_ + H_6′B_). ^13^C NMR (100 MHz, CDCl_3_) δ 13.7 (CH_3_, CH_3′_), 41.2 (CH_2_, CH_2′_), 55.9 (OCH_3_, OCH_3′_), 100.9, 110.8, 112.7, 115.3, 129.4, 130.4, 131.0, 131.5, 133.6, 138.2, 139.8, 156.5 (C_aryl_), 168.4 (Ph-C=O), 193.4 (Se-C=O). ^77^Se NMR (76 MHz, CDCl_3_) δ 657. HRMS *m/z*: calcd for C_38_H_30_Cl_2_N_2_O_6_Se_2_ [M + H]^+^, 840.9884; found, 840.9842. Purity (qNMR: 25 °C, CDCl_3_, m_s_ = 2.4 mg, m_IC_ = 4.0 mg): 96.6%.

*2-(6-Methoxynaphthalen-2-yl)propanoic diselenoperoxyanhydride* (**3**). The title compound was synthesized from (*S*)-2-(6-methoxy-2-naphthyl)propionyl chloride, selenium, and sodium borohydride according to the general procedure described above. A pink solid was obtained. Yield: 20%; mp: 113–115 °C. ^1^H NMR (400 MHz, CDCl_3_) δ 1.63 (d, 6H, *J* = 7.1 Hz, CH_3_, CH_3′_); 3.92 (s, 6H, OCH_3_, OCH_3′_); 4.17 (q, 2H, *J* = 7.1 Hz, CH, CH′); 7.13 (d, 2H, *J* = 2.3 Hz, H_5_, H_5′_); 7.16 (dd, 2H, *J* = 8.9 and 2.5 Hz, H_7_, H_7′_); 7.37 (dd, 2H, *J* = 8.6 and 1.7 Hz, H_8_, H_8′_); 7.73 (m, 6H, H_1_ + H_3_ + H_4_, H_1′_ + H_3′_ + H_4′_). ^13^C NMR (100 MHz, CDCl_3_) δ 18.3 (CH_3_, CH_3′_), 55.5 (OCH_3_, OCH_3′_), 56.6 (CH, CH′), 105.8, 119.5, 126.9, 127.8, 128.2, 129.0, 129.6, 133.3, 134.5, 158.3 (C_aryl_), 197.2 (Se-C=O). ^77^Se NMR (76 MHz, CDCl_3_) δ 636. HRMS *m/z*: calcd for C_28_H_26_O_4_Se_2_ [M + Na]^+^, 609.0054; found, 609.0061. Purity (qNMR: 25 °C, CDCl_3_, m_s_ = 1.8 mg, m_IC_ = 4.0 mg): 96.5%.

*2-(3-Benzoylphenyl)propanoic diselenoperoxyanhydride* (**4**). The title compound was synthesized from 2-(3-benzoylphenyl)propionic acid, oxalyl chloride, selenium, and sodium borohydride according to the general procedure described above. A white solid was obtained. Yield: 36%; mp: 65–66 °C. ^1^H NMR (400 MHz, CDCl_3_) δ 1.60 (dd, 6H, *J* = 7.1 and 1.5 Hz, CH_3_, CH_3′_); 4.16 (m, 2H, CH, CH′); 7.50 (m, 6H, H_3B_+H_4B_+H_5B_, H_3′B_+H_4′B_+H_5′B_); 7.59 (m, 4H, H_5A_ + H_6A_, H_5′A_ + H_6′A_); 7.79 (m, 8H, H_2A_ + H_4A_ + H_2B_ + H_6B_, H_2′A_ + H_4′A_ + H_2′B_ + H_6′B_). ^13^C NMR (100 MHz, CDCl_3_) δ 18.3 (CH_3_, CH_3′_), 56.4 (CH, CH′), 128.5, 128.6, 129.2, 130.2, 130.3, 130.4, 130.5, 132.6, 132.7, 132.8, 137.4, 138.4, 138.5, 138.6 (C_aryl_), 195.9 (Se-C=O), 196.3 (Ph-C=O). ^77^Se NMR (76 MHz, CDCl_3_) δ 633.8 and 634.1. HRMS *m/z*: calcd for C_32_H_26_O_4_Se_2_ [M + Na]^+^, 657.0054; found, 657.0062. Purity (qNMR: 25 °C, CDCl_3_, m_s_ = 1.7 mg, m_IC_ = 4.0 mg): 97.5%.

*2-(4-Isobutylphenyl)propanoic diselenoperoxyanhydride* (**5**). The title compound was synthesized from 2-(4-isobutylphenyl)propanoic acid, oxalyl chloride, selenium, and sodium borohydride according to the general procedure described above. A yellow oil was obtained. Yield: 44%. ^1^H NMR (400 MHz, CDCl_3_) δ 0.89 (d, 12H, *J* = 6.6 Hz, 2CH_3_, 2CH_3′_); 1.46 (d, 6H, *J* = 7.0 Hz, CH_3_, CH_3′_); 1.84 (sept, 2H, *J* = 6.8 Hz, CH, CH′); 2.44 (d, 4H, *J* = 7.2 Hz, CH_2_, CH_2′_); 3.98 (q, 2H, *J* = 7.2 Hz, CH-Ph, CH′-Ph); 7.09 (m, 8H, Hz, H_2_ + H_3_ + H_5_ + H_6_, H_2′_ + H_3′_ + H_5′_ + H_6′_). ^13^C NMR (100 MHz, CDCl_3_) δ 18.0, 18.1 (CH_3_, CH_3′_), 22.5 (2CH_3_, 2CH_3′_), 30.3 (CH, CH′), 45.2 (CH_2_, CH_2′_), 59.0, 59.1 (CH-Ph, CH′-Ph), 128.30, 128.33, 129.7, 135.28, 135.32, 141.57, 141.58 (C_aryl_), 198.6, 198.7 (Se-C=O). ^77^Se NMR (76 MHz, CDCl_3_) δ 791 and 792. HRMS *m/z*: calcd for C_26_H_34_O_2_Se_2_ [M + Na]^+^, 561.0781; found, 561.0748. Purity (qNMR: 25 °C, CDCl_3_, m_s_ = 3.0 mg, m_IC_ = 4.0 mg): 98.7%.

### 2.2. Biology

#### 2.2.1. DPPH Radical Scavenging Assay

The antioxidant activity of the novel diacyl diselenide-based compounds was determined by the 2,2-diphenyl-1-picrylhydrazyl (DPPH) colorimetric assay following a previously published procedure [22]. Briefly, the compounds were dissolved at a concentration of 1 mg/mL in absolute methanol, and a dilution was prepared. The stability of the DPPH radical through the time of analysis was checked by absorbance measurements of a methanolic solution (100 µM) of DPPH protected from light. Then, 100 µL of the DPPH solution was added to 100 µL of the compounds’ solution to a final concentration of 0.03 mg/mL or 0.06 mg/mL, and the decolorization of the purple radical to the yellowish reduced form was followed by recording the absorbance at 517 nm. Ascorbic acid (Asc) and Trolox were used as positive controls. Both of them, Asc and Trolox, were purchased from Sigma-Aldrich. Determinations were recorded at eight different time intervals from 0 to 120 min on a BioTeck PowerWave XS spectrophotometer, and the data were collected using KCJunior version 1.41. software. All the measurements were carried out in triplicate. Results are expressed as the percentage of the radical scavenged, calculated using the following formula: % DPPH radical scavenging = [(*A_control_* − *A_sample_*)/*A_control_*] × 100, wherein *A_control_* refers to the absorbance of the negative control and *A_sample_* refers to the absorbance of the tested compounds.

#### 2.2.2. Cell Culture Conditions

The cell lines were purchased from the American Type Culture Collection (ATCC). The cancer cell lines DU-145, PC-3, T-47D, MDA-MB-231, H1299, and A549, and the non-tumorigenic cells (184B5 and BEAS-2B), were maintained in RPMI 1640 medium (Gibco, Alcobendas, Madrid, Spain) supplemented with 10% fetal bovine serum (FBS; Gibco) and 1% antibiotics (10.00 units/mL penicillin and 10.00 mg/mL streptomycin; Gibco). Colon cancer cells HT-29 and HCT-116 were cultured in McCoy’s 5A medium (Gibco) supplemented with 10% fetal bovine serum (FBS; Gibco) and 1% antibiotics (10.00 units/mL penicillin and 10.00 mg/mL streptomycin; Gibco). Cells were maintained in tissue culture flasks at 37 °C and 5% CO_2_. The culture medium was replaced every three days.

#### 2.2.3. Cell Viability Assay

The antiproliferative activity and selectivity in vitro of the Se-NSAID derivatives were determined by the (3-(4,5-dimethylthiazol-2-yl)-2,5-diphenyltetrazolium bromide) (MTT) assay [37]. Compounds were dissolved in DMSO at a concentration of 0.01 M and serial dilutions were prepared. The Se-NSAID derivatives were tested at seven concentrations ranging between 1 and 100 µM in the colon (HT-29 and HCT-116), prostate (DU-145 and PC-3), breast (MDA-MB-231 and T-47D), and lung (H1299 and A549) cancer cell lines. The non-tumorigenic breast (184B5) and lung (BEAS-2B) cell lines were also tested to assess the selectivity of the compounds following the same procedure. A total of 1 × 10^4^ cells were seeded per well in 96-well plates and incubated at 5% CO_2_ and 37 °C for 24 h. Then, cells were treated with either DMSO or increasing concentrations of the corresponding Se-NSAID derivatives for 48 h. A volume of 20 µL of MTT (5 mg/mL) was added to each well 2.5 h before the termination point. At the end of the experiment, the medium was removed, and the resultant formazan crystals formed by mitochondrial reduction of MTT were dissolved in 50 µL of DMSO. Absorbance was measured at 550 nm. IC_50_, GI_50_, TGI, and LC_50_ values were calculated using OriginPro version 8.5.1. software by nonlinear curve fitting. Selectivity indexes (SI) were calculated as the ratio of the IC_50_ values determined for the nonmalignant and the tumoral cells in the breast (IC_50_ (184B5)/IC_50_ (MDA-MB-231) and IC_50_ (184B5)/IC_50_ (T-47D)) and lung (IC_50_ (BEAS-2B)/IC_50_ (H1299) and IC_50_ (BEAS-2B)/IC_50_ (A549)) cell lines. Data were obtained from at least three independent experiments performed in triplicates.

#### 2.2.4. Apoptosis Assays

The capacity of the Se-NSAID derivatives to induce apoptosis was analyzed using both a Caspase 3/7 assay kit and Annexin V & Dead Cell assay kit (EMD Millipore, Darmstadt, Germany). HCT-116 cells were seeded in 6-well plates at a density of 5 × 10^5^ cells per well. Cells were treated either with DMSO (control) or with different concentrations of compound **5** and incubated for 24 h. At the end of the treatment, cells were collected and treated with the respective dyes according to the manufacturer’s protocol. For the Caspase 3/7 assay, cells were stained with 5 µL of Muse™ Caspase 3/7 working solution and incubated at 37 °C for 30 min. After incubation, 150 mL of Muse™ Caspase 7-AAD working solution was added and the samples were incubated for another 5 min in the dark at room temperature prior to analysis. For Annexin V assay, cells were stained with 100 µL pf Muse™ Annexin V & Dead Cell Reagent and incubated for 20 min at room temperature in the dark. Samples for both assays were analyzed on a Muse™ Cell Analyzer (Merck Millipore, Darmstadt, Germany) using Muse™ version 1.4. software.

#### 2.2.5. In Vivo Xenograft Studies

Female mice of 4–5 weeks of age (The Jackson Laboratory, Bar Harbor, ME, USA) were kept on the daily cycle of 12 h light/dark and received food and water ad libitum throughout the studies. All animal procedures were performed following the National Institutes of Health (NIH) Guide for the Care and Use of Laboratory Animals and protocols were approved by the Animal Care and Use Committee of the Penn State College of Medicine.

#### 2.2.6. Toxicity Study

Mice were treated either with intraperitoneal doses of 7.5 mg/Kg of compound **5** or with DMSO (vehicle) thrice weekly and kept under observation for 27 days. Body weight, behavior, and food and water intake were monitored every alternate day. At the end of the study, animals were sacrificed by euthanizing the animals by CO_2_ asphyxiation. The blood serum was withdrawn and subjected to the biochemical analysis of creatinine (mg/dL), AST(SGOT) (U/L), ALT(SGPT) (U/L), Alk. Phos (U/L), and BUN (mg/dL).

#### 2.2.7. Assay of Tumor Growth Inhibition on HCT-116 Colon Cancer Xenograft Model

All mice were housed in quarantine for one week prior to the inoculation of the cancer cells. Female nude mice (4–5 weeks old) were subcutaneously injected (100 µL of PBS/flank) with 2.5 × 10^6^ HCT-116 colon cancer cells in each flank. Two weeks after inoculation, mice were sorted randomly into treatment and control groups (five mice/group) such that the mean tumor volumes were similar between groups. Mice from the treatment group were then injected intraperitoneally with 10 mg/Kg of compound **5** every 2 days over a 5-week treatment period. Mice that were injected with only DMSO (vehicle) were used as negative controls. The body weight of animals and the tumor volume were monitored thrice weekly. Tumor volumes were calculated using the formula (*L* × *W*^2^)/2, wherein *L* and *W* are the length and width of the tumor mass measured with a vernier caliper, respectively. At the end of the experiment, mice were sacrificed by euthanizing the animals by CO_2_ asphyxiation, and the tumors were excised and weighed. Inspection of major morphological changes in organs was also carried out. The antitumor effect was assessed by the tumor growth inhibition (TGI) and the relative increment ratio (T/C) parameters in the aspects of tumor volume and tumor weight, respectively. The TGI was calculated using the following formula: TGI (%) = [1 − (*T* − *T*_0_)/(*C* − *C*_0_)] × 100, wherein *T* and *C* are the final tumor volumes measured at the end of the treatment of compound-treated mice and vehicle-treated mice, respectively, whereas *T*_0_ and *C*_0_ are the tumor volumes measured at the beginning of the treatment of compound-treated mice and vehicle-treated mice, respectively. The T/C value was calculated as *W_T_*/*W_C_* × 100, wherein *W_T_* is the mean of tumor weight of the compound-treated mice, and *W_C_* is the mean of tumor weight of the vehicle-treated mice.

#### 2.2.8. Statistical Analysis

Data were expressed as the mean ± SD (standard deviation) unless otherwise specified, and experiments were performed at least thrice in triplicates. Non-linear curve regression analysis calculated by OriginPro version 8.5.1. software was used to assess the IC_50_, GI_50_, TGI, and LD_50_ values. For animal experiments, all animal studies were conducted using five animals per group (*n* = 5) and two tumors per animal. The two-way analysis of variance (ANOVA) was used to calculate the statistical significance of differences comparing compound **5** and control. Data were analyzed using GraphPad Prism version 8.0.1., and the statistically significant values (*p*-value) for ANOVA analysis were taken as **** *p* < 0.0001, ** *p* < 0.01, and * *p* < 0.05.

## 3. Results and Discussion

### 3.1. Structural Design

**Figure 2:**
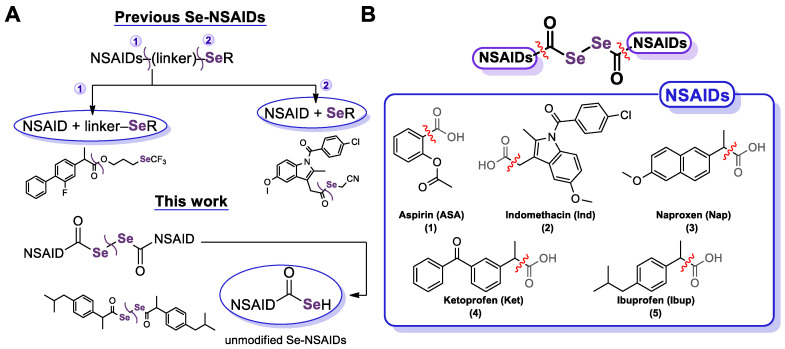
(**A**) Schematic diagrams of the chemical design of Se-modified NSAIDs, including previous possible cleavages according to where the bond is broken (1 or 2). (**B**) General structure of the new Se-NSAID derivatives **1**–**5** synthesized in this work and the parent NSAIDs used.

In recent years, the role of NSAIDs as single agents or in combination with current therapies in the prevention and treatment of cancer has attracted great interest, and their anticancer effect has been widely reported [41]. The modifications made to date in the literature reporting Se-NSAID analogs (Figure 1) considered the inclusion of the Se atom through well-known functional groups susceptible to being later metabolized to form active fragments. However, the part of the structure that retained the Se atom was never the NSAID itself, given the structural characteristics of these functional groups [60,61,62,63,64]. In this context, only selenocoxib-1 and selenocoxib-1-GSH [65] retained the Se moiety, but the NSAIDs were already modified and thus the parent celecoxib was not directly involved in the biological activity of the Se moiety once the compounds were metabolized. Consequently, there is currently no study reported to date regarding the biological activity of therapeutic Se-NSAID hybrids in which the entire NSAID could potentially be maintained without any structural modifications other than isosteric Se replacement of the oxygen atom of the NSAID-carboxylic acid group (Figure 2A). In this regard, given that the direct attempt to obtain the selenoic acid derivatives of NSAIDs could lead to unstable molecules, a bioisosteric replacement approach is proposed instead. Thus, we suggest that the modification of the NSAID scaffold via the novel inclusion of a diacyl diselenide functional group, which has recently been reported as carbonic anhydrase inhibitors [69], could lead to precursors for bioisosteric selenoic NSAID acids (Figure 2A). The rationale of our design resides in the liability and possible hydrolysis of the unique Se–Se bond present in the diacyl diselenide group. We hypothesized that this Se–Se bond would be liable to be broken in the metabolic process of the drug. Thus, the parent NSAID would retain the Se without any further modifications. Furthermore, the NSAID would also maintain the Se moiety in the form of a selenoic acid, in which -OH has been substituted with -SeH. SeH is a precursor of hydrogen selenide (H_2_Se), one of the main metabolites of Se [70] as it is the common intermediate of the reductive pathway and the catabolism of selenoamino acids [2] and with a prominent role in the biological activity of Se compounds [71].

Hence, five new selenocompounds were synthesized comprising diacyl diselenides decorated with two moieties of the corresponding NSAID forming a symmetric molecule, according to the general structure shown in Figure 2B. The NSAID scaffolds employed in the synthesis of the compounds covered a variety of chemical structures with reported chemotherapeutic activity, such as salicylate (ASA), acetic (indomethacin, Ind), and arylpropionic (naproxen, Nap, ketoprofen, Ket, and Ibup) acid derivatives. To our knowledge, this is the first time that a series of diacyl diselenide-based compounds has been reported to possess potent anticancer activity.

### 3.2. Chemistry

**Scheme 1:**
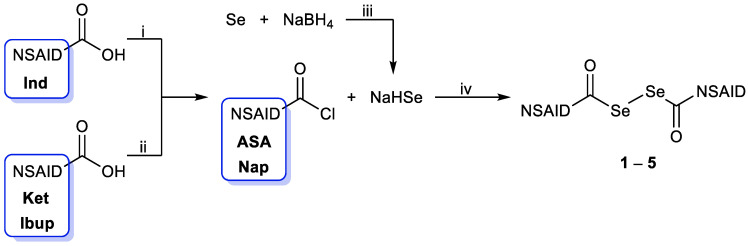
Synthesis of the Se-NSAID derivatives. Reagents and conditions: (i) ClCOCOCl, CH_2_Cl_2_, 12 h, room temperature; (ii) ClCOCOCl, CH_2_Cl_2_/*N*,*N*-DMF, 12 h, room temperature; (iii) H_2_O, room temperature; (iv) THF/H_2_O, 30–60 min, room temperature.

Some methods for obtaining diacyl diselenides have been reported previously. These methods include the reaction of acyl chlorides either with bis(trimethylsilyl)selenide (Me_3_SiSeSiH_2_Me_3_) [72]; with reduced Se from the reaction with samarium diiodide (SmI_2_) [73]; or with LiAlHSeH prepared from elemental Se and LiAlH_4_ and followed by quenching with the addition of I_2_ and KI [74,75,76]. The oxidation of potassium selenocarboxylates with xenon difluoride (XeF_2_) [77] has also been described, among others. However, these methods often include some inconveniences, such as the number of steps, the difficulty of purification, and the limited availability or expensive cost of some of the starting materials. Herein, we propose a new synthetic method for obtaining diacyl diselenide compounds in a single step by means of affordable reagents and using a non-contaminant solvent such as water. Thus, the syntheses of the novel Se derivatives of ASA, Ind, Nap, Ket, and Ibup were carried out by the reaction of the corresponding NSAID acyl chloride with an aqueous solution of sodium hydrogen selenide (NaHSe) formed in situ. In the case of NSAIDs Ind, Ket, and Ibup, the acyl chloride was not commercially available and had to be previously obtained from carboxylic acid. The synthesis route of these derivatives (**1**–**5**) is described in Scheme 1. The compounds were purified by silica gel column chromatography or flash chromatography with a gradient of hexane and ethyl acetate as eluent. All the diacyl diselenide NSAID derivatives were obtained with feasible yields (20–50%) in a one-pot synthesis.

The structures of all the compounds were confirmed by HRMS and by ^1^H, ^13^C, and ^77^Se NMR, as described in the previous section. The purity of all the compounds was determined by qNMR spectroscopy (see Supplementary Material). Concerning ^1^H NMR, a shift in the H peak in the groups close to the diacyl diselenide moiety could be observed due to the modification in the chemical environment of the carbonyl group. Thus, when the carbonyl of the diacyl diselenide moiety is attached to a methylene group as in the case of compound **2**, or to a methine group for the arylpropionic acid derivatives **3**, **4,** and **5**, a shift downfield of the corresponding H signal was found compared to the position of the same peak in the parent NSAID spectra. The methylene protons of compound **2** appeared at 4.00 ppm (Figure S6), whereas the same methylene attached to the carboxylic acid group of Ind appeared at 3.69 ppm. Likewise, the peak belonging to the single H of the methine group linked to the carbonyl appeared in the range of 3.98–4.17 ppm for compounds **3**, **4,** and **5**, while these signals appeared to be shifted upfield at 3.69–3.88 ppm in the ^1^H NMR spectra of the parent NSAIDs Nap, Ket, and Ibup, respectively, although the *J* values in all the cases were maintained at 7.2 Hz. Additionally, the two characteristic peaks of the ‘*para*’ substitution in the benzene ring of Ibup (7.09 and 7.21 ppm) merged into one signal at 7.09 ppm for compound **5** (Figure S15) marked as a multiplet. Inspection of ^13^C NMR spectra also revealed the displacement of the position of the group linked to the carbonyl of the diacyl diselenide moiety. As in the case of ^1^H NMR spectra, the peaks belonging to the methylene or the methine groups appeared to shift downfield when compared to the position of the signals in the NSAID spectra. For compound **2**, the methylene group appeared at 41.2 ppm (Figure S7), and this same peak appeared at 30.1 ppm in the case of Ind. The chemical environment of the methine group in Nap, Ket, and Ibup is highly similar, and so the signals in the corresponding ^13^C NMR spectra appeared almost in the same position, around 45.3 ppm. As expected, the incorporation of the diacyl diselenide moiety also modified the position of the methine in the same manner, these signals being shifted downfield in the range of 56.4–59.1 ppm. When comparing the new Se-NSAID derivatives with their corresponding parent NSAIDs, an evident shift downfield in the position of the carbonyl group attached to the double Se bond is observed. In this regard, the carboxylic acid groups of the parent NSAIDs appeared in the range of 170.3–181.1 ppm, ASA being the NSAID with the peak appearing as the most upfield. Accordingly, the carbonyl of the Se-NSAID derivatives showed an interval of 184.5–198.7 ppm, in which compound **1** (Figure S4) was the only derivative with the signal appearing below 190 ppm. This could probably be due to the aromatic ring attached to the carbonyl of **1** in comparison with the rest of the NSAID derivatives included in this study. Regarding ^77^Se NMR, overall, the Se peak for the diacyl diselenides appeared as one sharp peak in the range of 634–792 ppm. Interestingly, in the case of derivatives **4** (Figure S14) and **5** (Figure S17), two peaks located in close proximity to one another could be defined separately, corresponding to the two Se atoms of the double bond. These chemical shifts observed for the Se atom are in concordance with other ranges reported in the literature concerning diacyl diselenides [75]. Thus, compounds **3** and **4** showed a similar chemical shift for the Se peak in the range of 634–636 ppm, whereas the Se signals of derivatives **1** and **2** appeared slightly shifted downfield to 659 and 657 ppm, respectively, despite not having the same chemical environment as in the case of **3** and **4**. Likewise, the Se peaks of compound **5** appeared downfield at 791 and 792 ppm, although the chemical environment of the Se was expected to be similar to that of the other Se-NSAID derivatives of arylpropionic acids **3** and **4**.

### 3.3. Evaluation of the Radical Scavenging Activity of the Se-NSAID Derivatives

**Figure 3:**
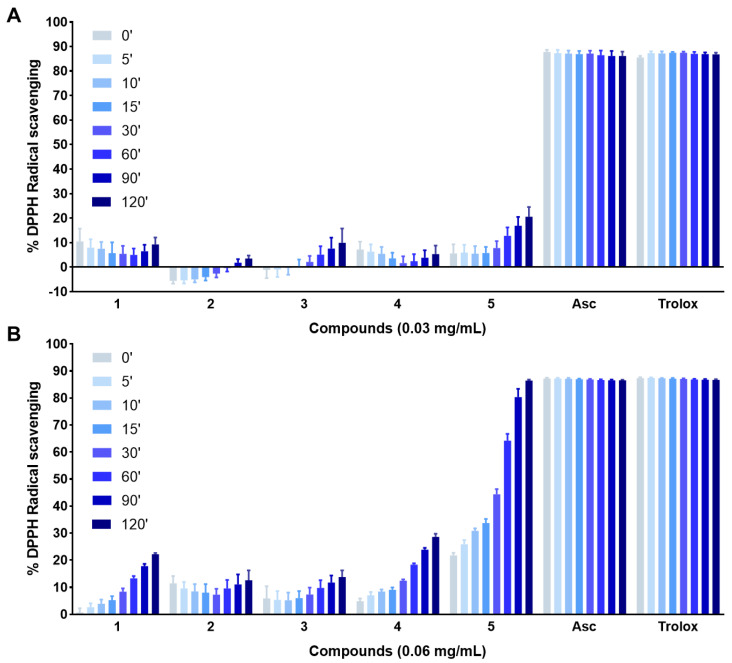
(**A**) DPPH radical scavenging activity of the Se-NSAID derivatives at 0.06 mg/mL and measured at eight time points. (**B**) DPPH radical scavenging activity of the Se-NSAID derivatives at 0.03 mg/mL and measured at eight time points. Data are presented as the mean ± SD of three independent experiments.

We first evaluated the radical scavenging capacity of the diacyl diselenides as a preliminary study to assess their potential antioxidant activity. Thus, the DPPH assay was performed for all the compounds at two different concentrations (0.06 and 0.03 mg/mL) and measured at eight different time points. Asc and Trolox were used as positive controls. The eight different time points and the two different concentrations were selected with the aim of achieving a significant DPPH radical scavenging activity for the diacyldiselenides. The same conditions, for comparison purposes, were used for the positive controls (Asc and Trolox), even though the maximal scavenging activity was demonstrated by both positive controls for each time point and concentration. The results obtained are summarized in Figure 3 and presented as the percentage of DPPH radical scavenging activity.

As shown in Figure 3, at the lowest concentration tested (Figure 3A), the compounds exhibited in general low activity, with the sole exception of compound **5** having an antioxidant capacity of 20%. Nevertheless, the Se-NSAID derivatives displayed an increase in their antioxidant activity over time, this effect is clearly observed at the highest concentration tested (Figure 3B). Thus, compounds **1**–**4** showed values of around 20–30% by the end of the experiment, surpassing the radical scavenging activity of other reported selenocompounds with therapeutic properties [22,66,78]. Remarkably, compound **5** was found to display the most potent radical scavenging activity among all the Se-NSAID derivatives, achieving values even comparable to those of the positive controls by the end of the assay (Figure 3B). Interestingly, no antioxidant activity could be ascribed to several selenocompounds that included the NSAID scaffolds [66], so the effect observed for these derivatives could be highly related to the presence of the diacyl diselenide group in their structures.

To the best of our knowledge, the results outlined in Figure 3 represent the first report of the antioxidant capacity of this type of molecule, therefore revealing the diacyl diselenide moiety as a new scaffold worth further exploration for developing compounds with antioxidant properties.

### 3.4. Evaluation of the Antiproliferative Activities of the Se-NSAID Derivatives

As a first approach to evaluate the possible anticancer properties of the new diacyl diselenides, the effect of the Se-NSAID derivatives on the cell viability was determined in vitro across a panel of cancer cell lines using the MTT assay as previously described [37]. The antiproliferative activity of compounds **1**–**5** was thereby assessed against two cell lines each of four different types of cancer (colon, prostate, breast, and lung) at seven concentrations ranging between 1 and 100 µM for 48 h. The calculated IC_50_ values are outlined in Table 1. The GI_50_, TGI, and LD_50_ values were also determined and are included in Table S1. The IC_50_ values obtained for each parent NSAID (ASA, Ind, Nap, Ket, and Ibup) are included for reference.

As reported in Table 1, compounds **1**–**5** displayed distinct antiproliferative profiles. Since the difference between compounds lies in the NSAID scaffold attached to the diacyl diselenide group at both sides of the molecule, it can be implied that the NSAID is a determinant for the antiproliferative activity. In this context, ASA derivative **1** showed potent antiproliferative activity in one of the colon cancer cell lines (HT-29) and in a breast cancer cell line (T-47D). Additionally, **1** showed IC_50_ values below 10 µM in both the prostate cancer cells (DU-145 and PC-3) and the lung cancer cells (H1299 and A549). In contrast, **1** presents relatively less antiproliferative effect towards triple-negative breast cancer cells (MDA-MB-231) and particularly to HCT-116 colon cancer cells, with an IC_50_ value of almost 40 µM. Compound **4** including a Ket scaffold displayed a similar profile as compound **1**, as it showed higher antiproliferative activity towards certain cell lines. In this case, **4** was active in at least one of the cell lines for the different types of cancer tested, with low IC_50_ values in HCT-116, PC-3, MDA-MB-231, and A549 cells. Interestingly, Ind derivative **2** showed no activity against all the eight cell lines evaluated, this being the only compound in which the inclusion of Se did not improve the antiproliferative capacity of the parent NSAID. The GI_50_, TGI, and LD_50_ values calculated also demonstrated poor activity of compound **2** in comparison with the rest of the series (Table S1).

Compound **3**, featuring a Nap scaffold, showed potent activity in both the prostate (DU-145 and PC-3) and breast (MDA-MB-231 and T-47D) cancer cell lines, with an average IC_50_ value of around 5 µM in these cells. On the contrary, this compound displayed selective activity in HCT-116 and H1299 cells, with no activity at all in the other cancer cells of the same type (HT-29 and A549, respectively). Ibup derivative **5** was the only compound among the Se-NSAID derivatives which displayed potent antiproliferative activity in all the cancer cells tested, with IC_50_ values below 10 µM in every colon, prostate, breast, and lung cancer cell line assessed. The GI_50_, TGI, and LD_50_ values obtained for this compound showed the same trend observed for the IC_50_ values (Table S1). Interestingly, only compounds **3** and **5** showed GI_50_ values below 10 µM across all the cancer cell lines tested, but compound **5** was the only Se-NSAID derivative with GI_50_ values even below 5 µM in at least six out of the eight cell lines.

Since unwanted side effects are usually associated with a lack of selectivity in the antiproliferative activity towards healthy cells, we then decided to test the Se-NSAID derivatives in mammary gland (184B5) and bronchial epithelium (BEAS-2B) nonmalignant cell lines. Thus, the selectivity indexes (SI) were determined as the ratio of the IC_50_ values obtained for the nonmalignant cells and the homolog breast and lung cancer cells. The IC_50_ values obtained in these nonmalignant cell lines and the calculated SI values are summarized in Table 2, together with the results already shown for MDA-MB-231, T-47D, H1299, and A549 cells for clarity. The GI_50_, TGI, and LD_50_ values were also calculated in the nonmalignant cells and are included in Table S1.

As shown in Table 2, compounds **1** and **5** showed the highest IC_50_ values among the Se-NSAID derivatives in the range of 14–19 µM in the nonmalignant cell lines. Hence, **1** showed SI values > 2 in the lung cells, but it lacked selectivity in breast cells when comparing the triple-negative breast cancer cell line (MDA-MB-231) with the nonmalignant cells (184B5). On the contrary, compound **5** displayed significant selectivity towards all the cell lines tested, with fair SI values of almost five- and four-fold in breast and lung cancer cells, respectively. Moreover, **5** was not only the best compound in terms of IC_50_ values in breast cancer cells, but it also inhibited cell growth without leading to cell death in breast nonmalignant cells (Table S1). On the other hand, similar to the antiproliferative activity tested in cancer cells, compound **2** was completely inactive in the two nonmalignant cell lines, and no IC_50_, GI_50_, TGI, and LD_50_ values could be determined (Table 2 and Table S1). Compound **3** was fairly selective in breast cells with SI values around two in both cases, but it showed poor selectivity in lung cancer cells, especially since this compound was not active in A549 cells. Compound **4** displayed IC_50_ values around 5 µM in the two nonmalignant cells, and thus was the less selective derivative among the compounds, mainly in lung cancer cells (SI values < 0.5). Therefore, compound **5** was identified as the most effective and selective Se-NSAID derivative overall.

Although the number of synthesized compounds is relatively small to make solid structure-activity relationships, some conclusions can be drawn from an overview analysis of the IC_50_ and SI values outlined in Table 1 and Table 2: (1) the Se atom incorporated in the novel form of a diacyl diselenide group into NSAID derivatives is a valid approach to obtain potent antiproliferative compounds; (2) the NSAID is also determinant for the biological activity, as the presence of an Ind scaffold at both sides of the diacyl diselenide group resulted in an inactive compound **2** with no activity in the panel of cancer cell lines tested; (3) NSAID Ibup yielded the best NSAID derivative in terms of both the activity and selectivity, as it stood out with a good selectivity towards cancer cells while evincing the most potent antiproliferative activity in all the cancer cell lines tested.

Considering the selectivity and anticancer activity displayed by compound **5**, this Ibup derivative was selected for further biological studies. This compound showed outstanding antiproliferative activity with IC_50_ values below 10 µM in all the cancer cell lines evaluated (Table 1). Additionally, **5** proved to be the most selective compound among all the Se-NSAID derivatives (Table 2). Interestingly, compound **5** also displayed the most potent antioxidant activity in the DPPH radical scavenging assay (Figure 3), thus suggesting a dual behavior for this analog.

### 3.5. Compound ***5*** Induced Cell Death by Partially Triggering Apoptosis in Colon Cancer Cells

The assays performed on cell viability evinced that compound **5** showed potent antiproliferative activity. Thus, we evaluated this derivative to further confirm if **5** induced cell death through apoptosis. Since sequential activation of caspases is essential for beginning the process of apoptosis [79], we tested the effect of compound **5** on caspase-3 and caspase-7 with the Caspase 3/7 assay. The Annexin V & Dead Cell assay was also performed to complement the results.

**Figure 4:**
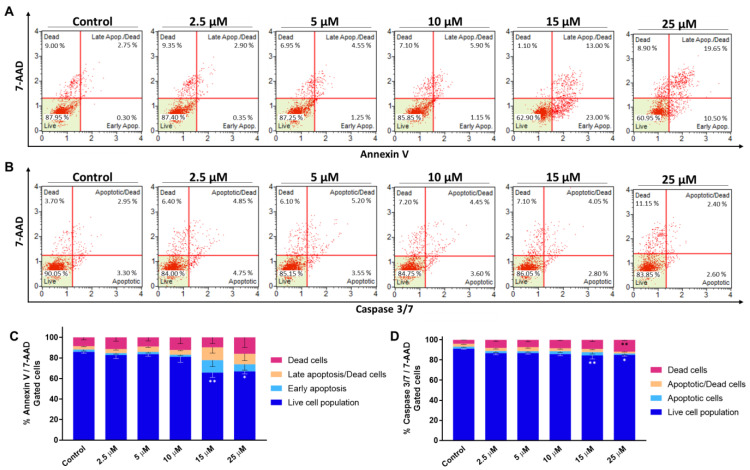
Induction of apoptosis in HCT-116 cells. (**A**) Cells were treated with five concentrations of **5** for 24 h and examined on a Muse™ automated cell analyzer with the Annexin V & Dead Cell apoptosis assay. (**B**) Analogous independent experiment performed with the Caspase 3/7 apoptosis assay. (**C**,**D**) Quantification of the cell population with the Annexin V & Dead Cell and Caspase 3/7 assays, respectively. Data are presented as the mean ± SEM of at least three independent experiments. ** *p* < 0.01, * *p* < 0.05 comparing control and different concentrations of compound **5**.

NSAIDs have been widely studied and their chemopreventive activity in colon cancer has been well documented in the literature [48,49]. Several studies also reported their therapeutic effect and their mechanism of action in this cancer [47,58,80,81], which encouraged us to select the colon cancer HCT-116 cell line as representative cells to focus our experiments on. As shown in Table 1 and Table S1, compound **5** showed potent antiproliferative activity in all the cancer cell lines tested, including HCT-116. Therefore, HCT-116 cells were treated either with DMSO (vehicle) or compound **5** for 24 h at five different concentrations ranging from 2.5 to 25 µM. The results were obtained following the manufacturer’s protocol and are shown in Figure 4. In addition, a quantitative comparison of the difference in the cell population induced by compound **5** is included in Figure 4C,D.

In general, cells treated only with DMSO were located as expected mainly in the lower left quadrant in both assays. The Annexin V & Dead Cell assay evinced that the treatment with compound **5** induced a shift from healthy cells towards an apoptotic state (Figure 4A,C). However, even at a concentration close to the IC_50_ value (10 µM), the cells remained mainly located in the lower left quadrant after 24 h and no discernible difference was observed compared to the control cells. As the concentration increased up to 15 µM, the live cell population decreased to ~63%, while the percentage of early apoptotic cells was consequently increased to ~23%. The population of cells in a late apoptosis state was also enhanced to ~13% (Figure 4A). Interestingly, at 25 µM, the percentage of viable cells treated with **5** was maintained at a value close to ~60%, while more than 30% of cells were detected to be apoptotic. In this context, a slight population of cells shifted to a necrotic or late apoptotic state, as there were ~10% or ~20% of cells, respectively, with 7-AAD positive (Figure 4A). Nevertheless, the presence of outer PS residues determined by the Annexin V & Dead Cell assay seems to be insufficient to explain the potent antiproliferative capacity observed in the cell viability assays (Table 1 and Table S1).

Considering the Caspase 3/7 assay (Figure 4B,D), the percentage of the live cell population was maintained even at high concentrations of compound **5** with almost no variations, although at 15 and 25 µM a significant change could be observed (Figure 4D). There was only a slight increase in the population of dead cells up to ~11% eventually at 25 µM, but the distribution of cells in an apoptotic state was not significantly different with respect to the control cells treated only with DMSO. Thus, these results imply that compound **5** might not exert its anticancer activity through significant involvement in the caspase 3/7 activity.

Therefore, the results shown in Figure 4, along with the anticancer activity displayed by compound **5** in HCT-116 cells (Table 1), could suggest that apoptosis is not the only mechanism by which this compound induces cell death in this type of cancer cells.

### 3.6. Compound ***5*** Effectively Inhibited Tumor Growth in a Colon Cancer Xenograft Model without Any Apparent Systemic Toxicity

The overall appealing profile of compound **5** regarding its antiproliferative activity in vitro prompted us to further assess its toxicity and efficacy in vivo.

**Figure 5:**
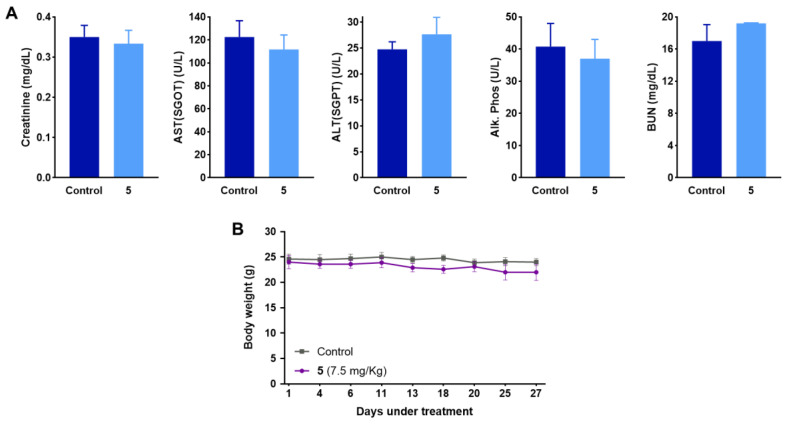
In vivo toxicity of compound **5** in mice. Female nude mice (*n* = 5 in each group) were treated either with the vehicle or compound **5** for 27 days. (**A**) Biochemical analysis of the blood serum withdrawn at the endpoint. The treatment with a dose of 7.5 mg/Kg of **5** given thrice weekly showed no significant changes in the parameters measured compared to the control group. (**B**) Body weight changes of mice during the treatment. Data are presented as the mean ± SEM.

For chronic toxicity assessment, the mice were given a dose of **5** (7.5 mg/Kg body weight) intraperitoneally (IP) thrice per week, and related changes in body weight, behavior, food, and water uptake were monitored for almost a month. The blood serum was also collected after the sacrifice and analyzed. The results are displayed in Figure 5. As shown in Figure 5A, the blood biochemistry profile measured at the end of the treatment with **5** revealed no significant changes in the compound-treated mice compared to the vehicle-treated animals. Moreover, the body weight of the mice was maintained with no appreciable variations during the treatment with **5** (Figure 5B) and we observed no changes in behavior and food/water uptake.

**Figure 6:**
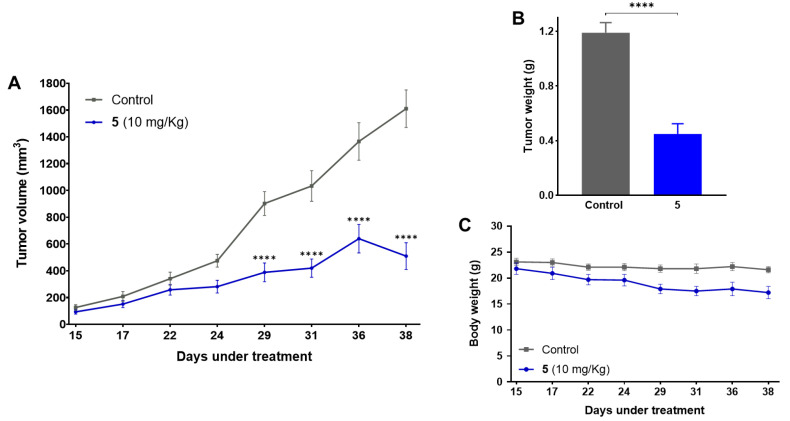
In vivo efficacy of compound **5** in a colon cancer xenograft mouse model. Female nude mice were inoculated with 2.5 × 10^6^ HCT-116 cells on each flank. After 15 days, the HCT-116 tumor-bearing mice (*n* = 5 in each group) were treated either with the vehicle or compound **5** for additional 23 days. (**A**) Growth curves of tumor volume. The treatment with a dose of 10 mg/Kg of **5** given thrice weekly achieved significant tumor growth inhibition compared to the control group. (**B**) Tumor weight measured at the endpoint. (**C**) Body weight changes of mice during the treatment. Data are presented as the mean ± SEM. **** *p* < 0.0001 comparing control and treatment with compound **5**.

The low toxicity of compound **5** warranted the evaluation of its antitumor activity in a subcutaneous xenograft tumor mouse model bearing HCT-116 colon cancer cells. Nude mice were randomized and treated IP with compound **5** (10 mg/Kg body weight) for 23 days. The results of the in vivo efficacy assay are outlined in Figure 6. A higher dose for the efficacy study in vivo was chosen based on the favorable results at 7.5 mg/Kg (Figure 5). The TGI and the T/C parameters were calculated at the end of the treatment to assess the antitumor effects in terms of tumor volume and tumor weight, respectively.

As shown in Figure 6, compound **5** significantly inhibited tumor growth in the colon cancer xenograft model. The pronounced inhibitory effect was maintained throughout the duration of the treatment (Figure 6A), and on day 38 the TGI reached 72% compared to the vehicle-treated mice. Furthermore, the comparison of the weight of the tumors excised at the end of the treatment (Figure 6B) with a good T/C ratio of 38% also evinced the significant inhibition of tumor growth by compound **5**. Besides, a slight change in the body weight of the mice was observed in the **5**-treated group compared to the vehicle-treated mice during the treatment (Figure 6C), but no evident toxicity signs as morphological abnormalities in major organs were detected. Taken together, our in vivo results demonstrated that compound **5** remarkably produced a significant inhibition of the tumor growth in a colon cancer xenograft model with no noticeable toxicity, suggesting that **5** is a promising candidate worthy of further preclinical studies. Nevertheless, the in vivo efficacy of compound **5** seems to present a limited off-target effect considering its different behavior in vitro and in vivo. This discrepancy could be attributed to several factors, such as instability of the selenocompound or inadequate ADME properties. Thus, different approaches can be explored to further develop this new class of anticancer agents, including structural modifications to improve solubility and biodistribution features, or employing a targeted delivery to both increase the stability and ADME properties.

## 4. Conclusions

In summary, we synthesized for the first time a small library of NSAID derivatives containing Se in the novel form of a diacyl diselenide group as precursors with the aim of retaining the Se moiety in the NSAID scaffolds. The work presented here evinces that this design based on the incorporation of Se as diacyl diselenide moieties into traditional NSAIDs, led to analogs with potent antioxidant and/or anticancer activity. To our knowledge, this constitutes the first report of the appealing in vitro and in vivo therapeutic profile of diacyl diselenide-based compounds. The results described herein demonstrate that our design approach to maintain the entire NSAIDs along with Se provides a feasible new frame to develop novel biologically active agents. Compound **5** was identified as the most potent antiproliferative agent among the Se-NSAID derivatives with IC_50_ values below 10 µM in a panel of eight cancer cell lines in vitro. Furthermore, **5** proved to be the most selective analog when compared to the nonmalignant cell lines. This compound also displayed the most potent antioxidant activity within the series, achieving values comparable to those of the positive controls as demonstrated in the DPPH radical scavenging assay. The capability of **5** to induce cell death through apoptosis was also evaluated in HCT-116 colon cells. Interestingly, no clear enhancement on the caspase 3/7 activity was observed, but **5** was found to partially induce apoptosis determined by an Annexin V assay. Notably, compound **5** also markedly inhibited the tumor growth in an HCT-116 colon cancer xenograft model at a dose of 10 mg/Kg administrated IP, with an impressive TGI value of 72% and a T/C value of 38%. Overall, these findings qualify compound **5** and the diacyl diselenide functionality as appealing candidates to explore the potential and utility of diacyl diselenide-based derivatives as leads to design precursors for bioisosteric selenocompounds as therapeutic agents, especially in colon cancer treatment.

## Figures and Tables

**Table 1:** IC_50_ values (in µM) ^1^ for the Se-NSAID analogs and parent NSAIDs in colon (HT-29, HCT-116), prostate (DU-145, PC-3), breast (MDA-MB-231, T-47D), and lung (H1299, A549) cancer cell lines.

Comp.	Colon Cell Lines		Prostate Cell Lines		Breast Cell Lines		Lung Cell Lines	
	HT-29	HCT-116	DU-145	PC-3	MDA-MB-231	T-47D	H1299	A549
**1**	6.6 ± 2.2	39.2 ± 7.3	5.0 ± 0.1	3.3 ± 0.6	18.3 ± 6.9	5.9 ± 1.4	8.6 ± 0.8	7.0 ± 1.3
**2**	>50	>50	>50	>50	>50	>50	>50	>50
**3**	>50	9.1 ± 5.9	6.1 ± 0.6	5.2 ± 0.2	5.7 ± 0.8	5.6 ± 0.1	8.1 ± 2.5	>50
**4**	39.7 ± 3.3	5.9 ± 1.1	27.9 ± 3.2	5.8 ± 0.6	4.3 ± 1.2	26.0 ± 2.0	31.6 ± 1.7	11.7 ± 0.9
**5**	8.5 ± 2.7	9.0 ± 3.0	7.0 ± 1.3	7.4 ± 0.5	8.3 ± 2.5	3.8 ± 0.2	9.3 ± 0.6	4.5 ± 0.6
**ASA ^2^**	>50	>50	>50	>50	>50	>50	>50	>50
**Ind**	>50	>50	>50	>50	>50	>50	>50	>50
**Nap**	>50	>50	>50	>50	>50	>50	>50	>50
**Ket**	>50	>50	>50	>50	>50	>50	>50	>50
**Ibup**	>50	>50	>50	>50	>50	>50	>50	>50

^1^ IC_50_ values are presented as the mean ± SD of at least three independent experiments determined by the MTT assay. ^2^ Some IC_50_ values for parent NSAIDs were obtained from Plano et al. [61], He et al. [62,63], and Ramos-Inza et al. [66].

**Table 2:** IC_50_ values (in µM) ^1^ for the Se-NSAID analogs in breast (MDA-MB-231, T-47D) and lung (H1299, A549) cancer cell lines along with the IC_50_ values (in µM) obtained in nonmalignant cells (184B5 and BEAS-2B) and the respective SI calculated.

Comp.	Breast Cell Lines					Lung Cell Lines				
	184B5	MDA-MB-231	SI ^2^	T-47D	SI ^3^	BEAS-2B	H1299	SI ^4^	A549	SI ^5^
**1**	14.4 ± 1.5	18.3 ± 6.9	0.8	5.9 ± 1.4	2.4	18.5 ± 3.0	8.6 ± 0.8	2.2	7.0 ± 1.3	2.6
**2**	>100	>100	-	>100	-	>100	>100	-	>100	-
**3**	11.9 ± 1.0	5.7 ± 0.8	2.1	5.6 ± 0.1	2.1	11.2 ± 1.0	8.1 ± 2.5	1.4	>100	>0.1
**4**	5.9 ± 0.8	4.3 ± 1.2	1.4	26.0 ± 2.0	0.2	5.2 ± 0.4	31.6 ± 1.7	0.2	11.7 ± 0.9	0.4
**5**	18.2 ± 0.8	8.3 ± 2.5	2.2	3.8 ± 0.2	4.8	15.9 ± 2.4	9.3 ± 0.6	1.7	4.5 ± 0.6	3.5

^1^ IC_50_ values are presented as the mean ± SD of at least three independent experiments determined by the MTT assay. ^2^ SI calculated in breast cells as IC_50_ (184B5)/IC_50_ (MDA-MB-231). ^3^ SI calculated in breast cells as IC_50_ (184B5)/IC_50_ (T-47D). ^4^ SI calculated in lung cells as IC_50_ (BEAS-2B)/IC_50_ (H1299). ^5^ SI calculated in lung cells as IC_50_ (BEAS-2B)/IC_50_ (A549).

## Data Availability

The data presented in this study are available upon reasonable request from the corresponding authors.

## Supplementary Materials

The following supporting information can be downloaded at https://www.mdpi.com/article/10.3390/antiox12091666/s1, Table S1: GI_50_, TGI and LD_50_ values; Figures S1–S20: ^1^H, ^13^C and ^77^Se NMR spectra, qNMR spectra; Figure S21: Mass spectrum of compound **5**.

## Abbreviations

ASA, aspirin; Asc, ascorbic acid; ATCC, American Type Culture Collection; COX, cyclooxygenase; DPPH, 2,2-diphenyl-1-picrylhydrazyl; FAP, familial adenomatous polyposis; FBS, fetal bovine serum; HRMS, high-resolution mass spectrometry; Ibup, ibuprofen; Ind, indomethacin; IP, intraperitoneally; Ket, ketoprofen; MMR, DNA mismatch repair; mp, melting point; MTT, 3-(4,5-dimethylthiazol-2-yl)-2,5-diphenyltetrazolium bromide; Nap, naproxen; NMR, nuclear magnetic resonance; NSAIDs, nonsteroidal anti-inflammatory drugs; PG, prostaglandin, Se, selenium; SI, selectivity index; T/C, relative increment ratio; TGI, tumor growth inhibition; TLC, thin-layer chromatography.
