# Supplementary material for: First Generation of Antioxidant Precursors for Bioisosteric Se-NSAIDs: Design, Synthesis, and In Vitro and In Vivo Anticancer Evaluation
Source: Antioxidants (Basel). 2023 Aug 24;12(9):1666. doi: 10.3390/antiox12091666 (PMC10525927; doi:10.3390/antiox12091666)
Supplement: Supplementary file 1 [file antioxidants-12-01666-s001.zip › antioxidants-2539382-supplementary.pdf]

First Generation of Antioxidant Precursors for Bioisosteric Se-NSAIDs: Design, Synthesis and In Vitro and In Vivo Anticancer Evaluation

Sandra Ramos-Inza [1,2], Cesar Aliaga [3], Ignacio Encío [4], Asif Raza [3], Arun K. Sharma [3,*], Carlos Aydillo [1,2,], Nuria Martínez [1], Carmen Sanmartín [1,2,*] and Daniel Plano [1,2,*]

[1] Department of Pharmaceutical Technology and Chemistry, University of Navarra, Irunlarrea 1, 31008 Pamplona, Spain
[2] Instituto de Investigación Sanitaria de Navarra (IdiSNA), Irunlarrea 3, 31008 Pamplona, Spain
[3] Department of Pharmacology, Penn State Cancer Institute, CH72, Penn State College of Medicine, 500 University Drive, Hershey, PA 17033, USA
[4] Department of Health Sciences, Public University of Navarra, Avda. Barañain s/n, 31008 Pamplona, Spain
* Correspondence: asharma1@pennstatehealth.psu.edu (A.K.S.); sanmartin@unav.es (C.S.); dplano@unav.es (D.P.)

Table of Contents

**Biological evaluation**		
**GI_{50}, TGI and LD_{50} values**	....	**Table S1**
**Chemical characterization**		
**^{1}H, ^{13}C and ^{77}Se NMR spectra, qNMR spectra**		
**1**	....	**Figures S1-S4**
**2**	....	**Figures S5-S8**
**3**	....	**Figures S9-S12**
**4**	....	**Figures S13-S16**
**5**	....	**Figures S17-S20**
**Mass spectrum of compound 5**	....	**Figure S21**

Biological evaluation

GI$_{50}$, TGI and LD$_{50}$ values

Table S1. GI$_{50}$, TGI and LD$_{50}$ values (in μM)[a] for the Se-NSAID analogs in colon (HT-29, HCT-116), prostate (DU-145, PC-3), breast (MDA-MB-231, T-47D, 184B5) and lung (H1299, A549, BEAS-2B) cell lines.

Compounds		Colon cell lines		Prostate cell lines		Breast cell lines			Lung cell lines		
		**HT-29**	**HCT-116**	**DU-145**	**PC-3**	**MDA-MB-231**	**T-47D**	**184B5**	**H1299**	**A549**	**BEAS-2B**
**1**	**GI$_{50}$**	3.9 ± 0.4	21.0 ± 4.0	4.4 ± 0.5	2.8 ± 0.2	6.6 ± 2.2	1.6 ± 0.3	7.0 ± 1.1	7.7 ± 1.2	>100	3.9 ± 1.3
	**TGI**	6.2 ± 1.9	44.5 ± 7.1	5.1 ± 0.2	3.0 ± 0.3	15.3 ± 4.6	2.9 ± 0.6	11.3 ± 1.4	9.4 ± 0.8	26.2 ± 5.8	8.4 ± 2.4
	**LD$_{50}$**	>100	88.0 ± 3.5	6.1 ± 0.7	3.3 ± 0.4	>100	6.0 ± 1.9	21.1 ± 3.9	>100	74.6 ± 5.9	17.0 ± 5.7
**2**	**GI$_{50}$**	>100	44.5 ± 3.4	58.4 ± 8.4	>100	23.1 ± 14.3	25.5 ± 10.2	>100	53.2 ± 1.2	>100	>100
	**TGI**	>100	81.5 ± 2.3	>100	>100	>100	39.0 ± 8.5	>100	>100	>100	>100
	**LD$_{50}$**	>100	>100	>100	>100	>100	>100	>100	>100	>100	>100
**3**	**GI$_{50}$**	6.8 ± 1.4	9.3 ± 4.7	5.0 ± 0.2	4.4 ± 0.3	5.2 ± 0.2	1.8 ± 0.2	7.1 ± 0.5	6.7 ± 1.1	3.3 ± 0.4	7.0 ± 2.5
	**TGI**	>100	30.9 ± 16.7	6.1 ± 0.6	5.0 ± 0.1	5.6 ± 0.8	3.1 ± 0.3	9.4 ± 0.2	>100	>100	9.0 ± 1.2
	**LD$_{50}$**	>100	>100	10.4 ± 2.4	5.9 ± 0.9	>100	6.3 ± 0.7	>100	>100	>100	12.3 ± 2.1
**4**	**GI$_{50}$**	24.4 ± 2.6	4.3 ± 0.6	19.5 ± 3.5	4.8 ± 0.4	2.4 ± 0.5	>100	4.8 ± 0.3	24.6 ± 1.8	8.1 ± 0.4	4.2 ± 0.6
	**TGI**	36.2 ± 3.0	6.7 ± 1.9	28.5 ± 2.9	5.5 ± 0.5	4.2 ± 1.7	9.5 ± 2.3	5.4 ± 0.4	33.6 ± 1.6	19.9 ± 3.4	4.7 ± 0.3
	**LD$_{50}$**	62.5 ± 14.3	>100	39.6 ± 2.4	6.7 ± 1.2	>100	27.2 ± 2.3	>100	43.5 ± 1.8	>100	5.4 ± 0.4
**5**	**GI$_{50}$**	4.8 ± 1.0	4.6 ± 1.5	6.0 ± 1.3	4.7 ± 0.2	4.8 ± 1.1	1.8 ± 0.4	6.6 ± 1.5	8.1 ± 1.0	2.3 ± 0.2	7.8 ± 2.3
	**TGI**	7.2 ± 1.8	10.3 ± 3.8	7.3 ± 0.9	6.1 ± 0.5	7.9 ± 3.8	2.7 ± 0.3	11.1 ± 1.4	10.2 ± 1.0	>100	9.8 ± 1.6
	**LD$_{50}$**	>100	>100	9.0 ± 0.4	8.9 ± 1.9	>100	4.2 ± 0.3	>100	16.5 ± 5.1	>100	14.8 ± 4.7

[a]GI$_{50}$, TGI and LD$_{50}$ values are presented as the mean ± SD of at least three independent experiments determined by the MTT assay.

௸ Chemical characterization – NMR spectra

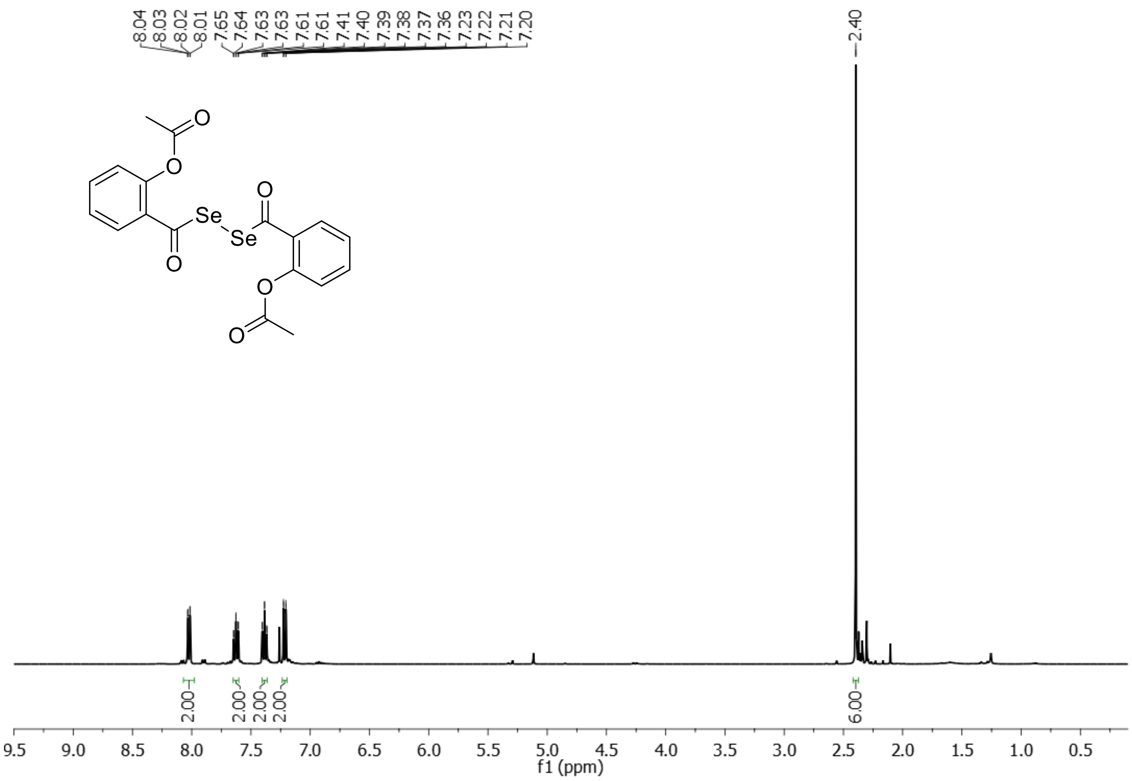

Figure S1. ^{1}H-NMR spectrum of compound **1**.

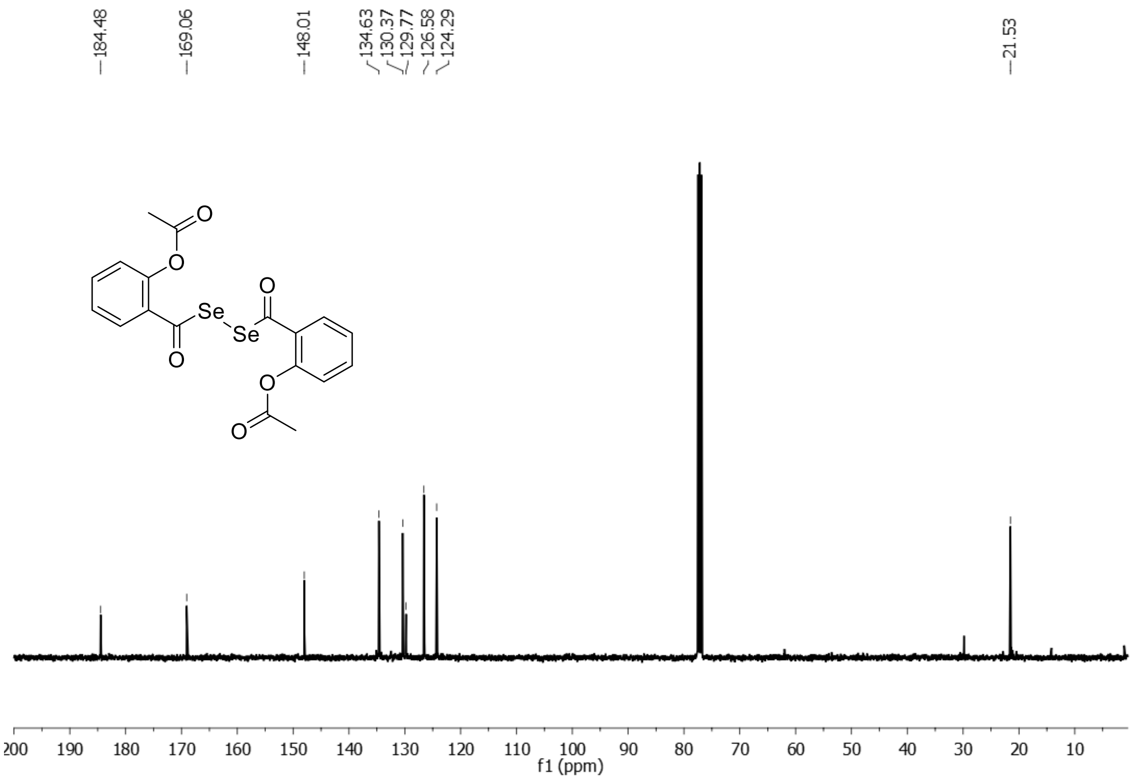

Figure S2. ^{13}C-NMR spectrum of compound **1**.

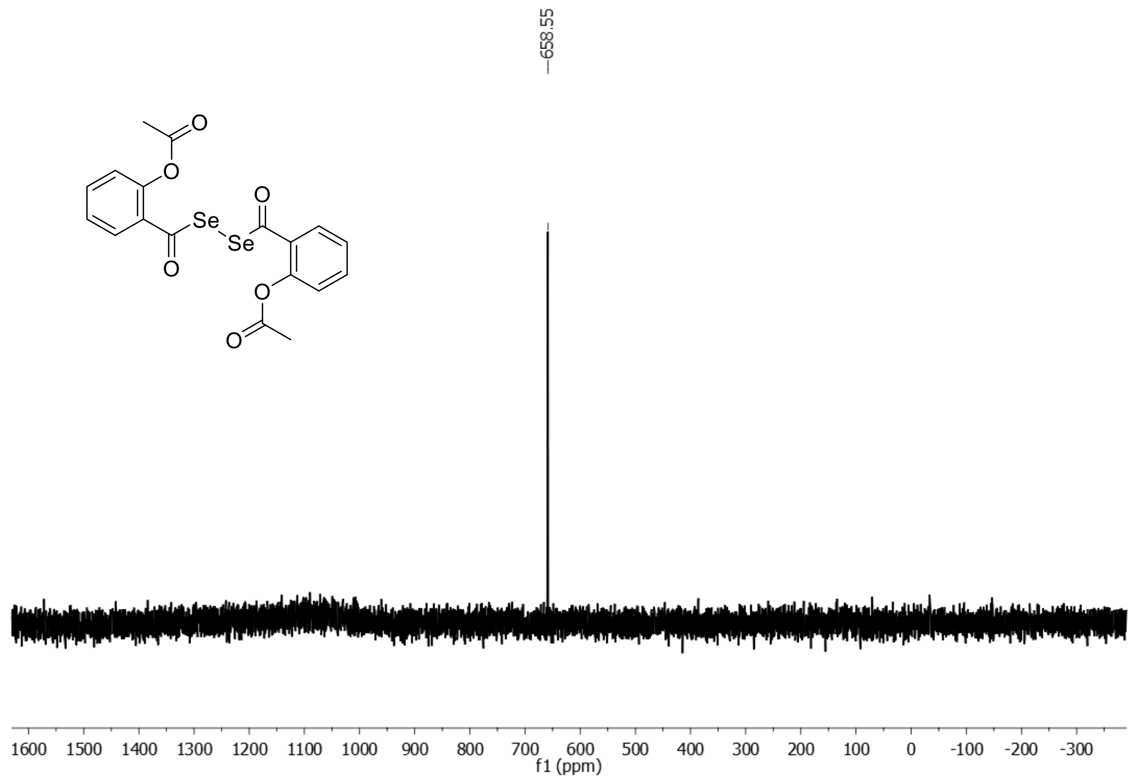

Figure S3. ^{77}Se-NMR spectrum of compound **1**.

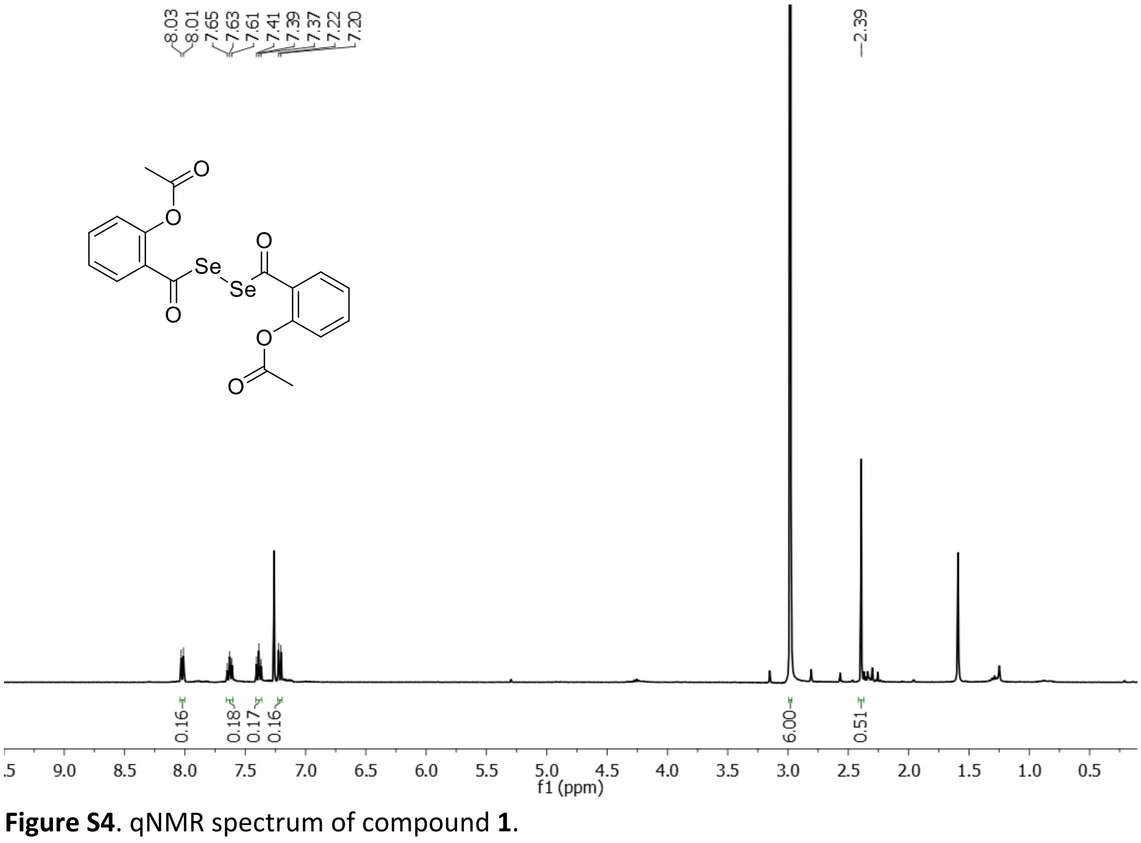

Figure S4. qNMR spectrum of compound **1**.

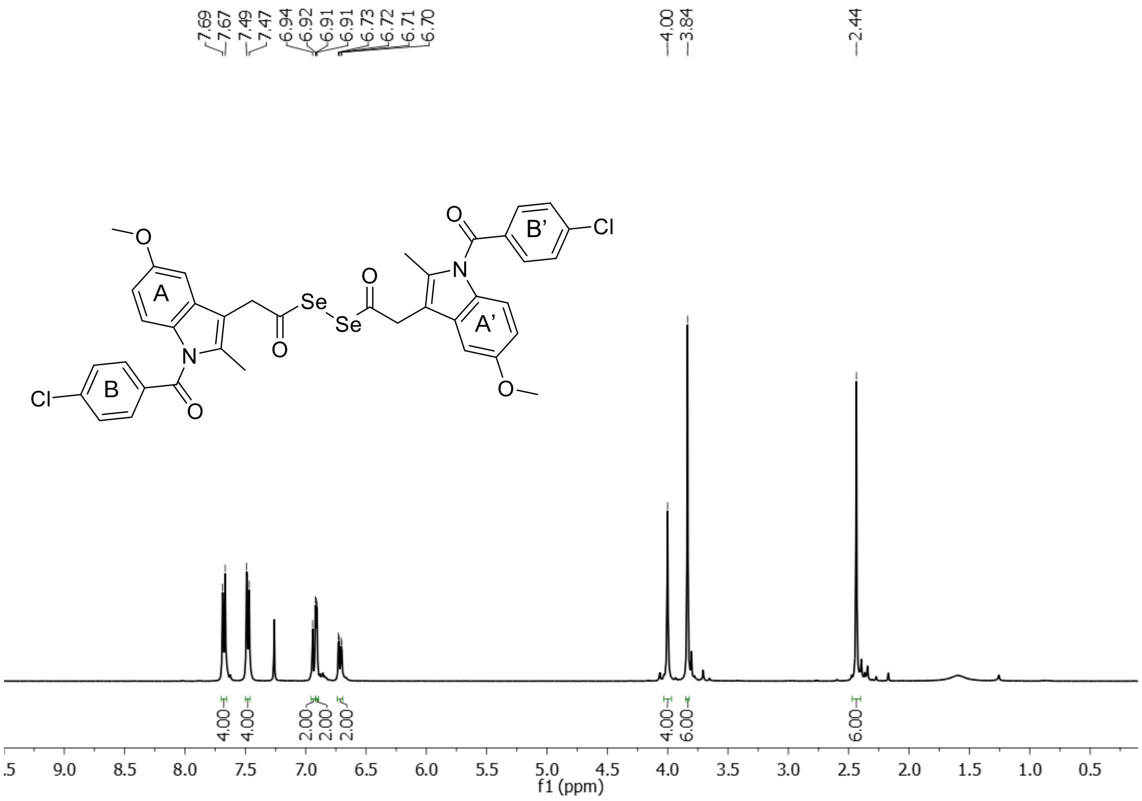

Figure S5. ^{1}H-NMR spectrum of compound **2**.

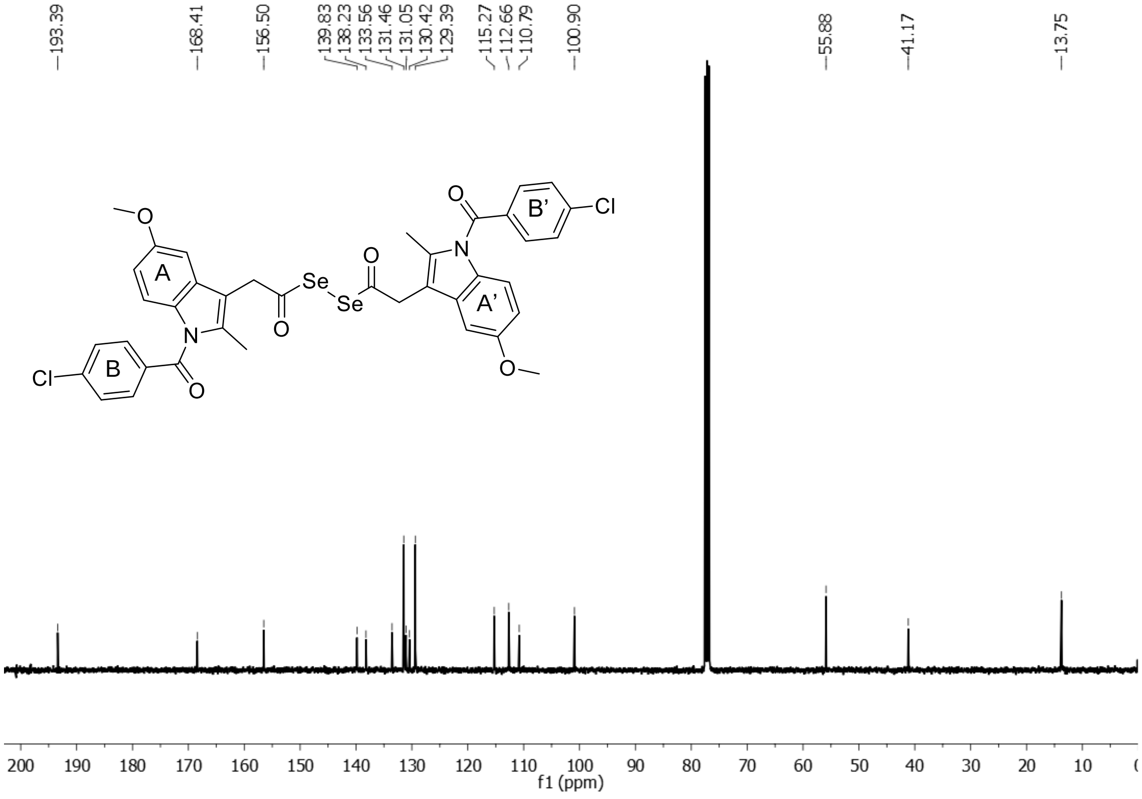

Figure S6. ^{13}C-NMR spectrum of compound **2**.

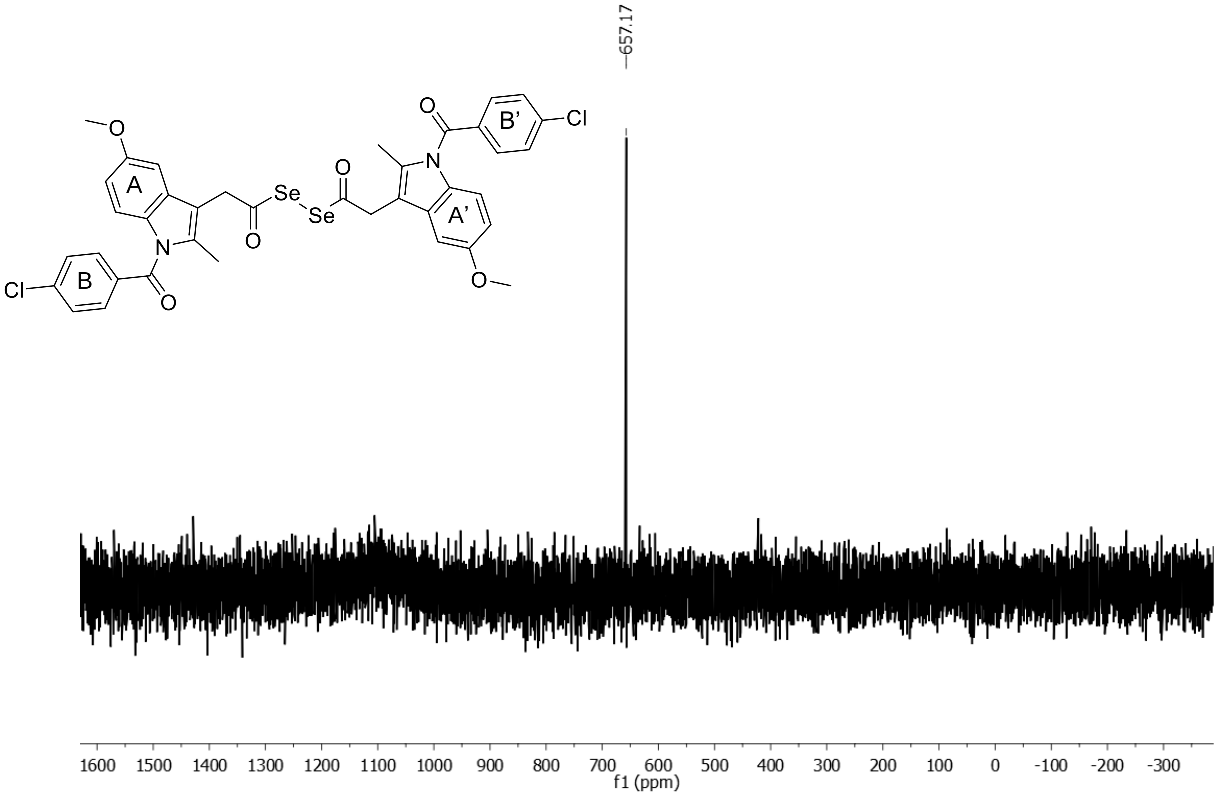

Figure S7. ^{77}Se-NMR spectrum of compound **2**.

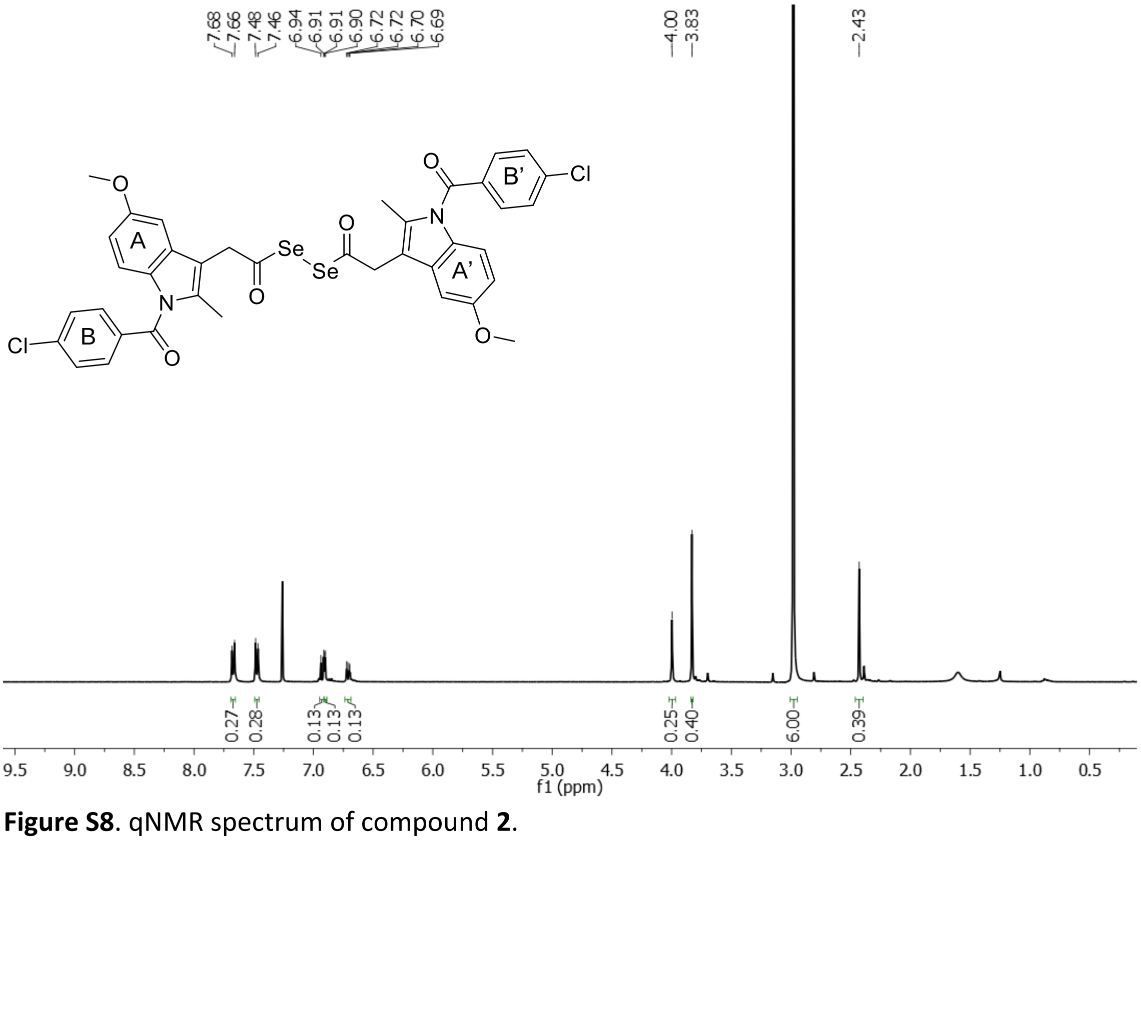

Figure S8. qNMR spectrum of compound **2**.

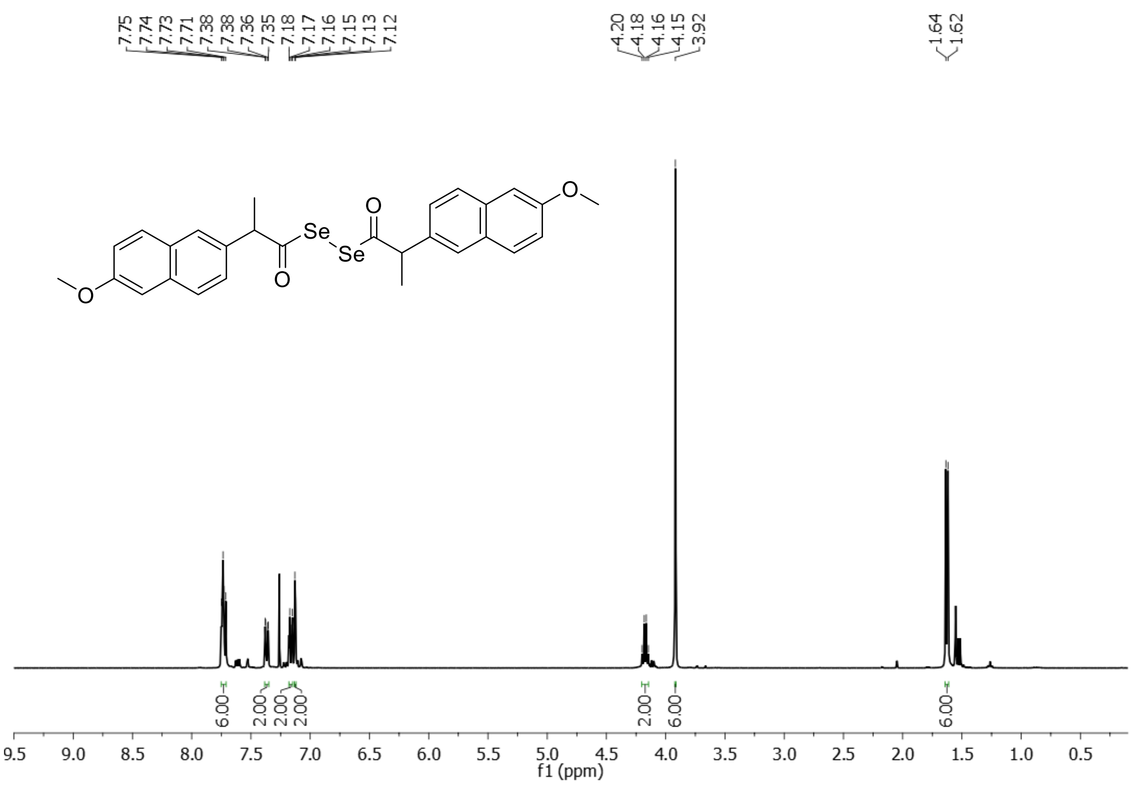

Figure S9. ^{1}H-NMR spectrum of compound **3**.

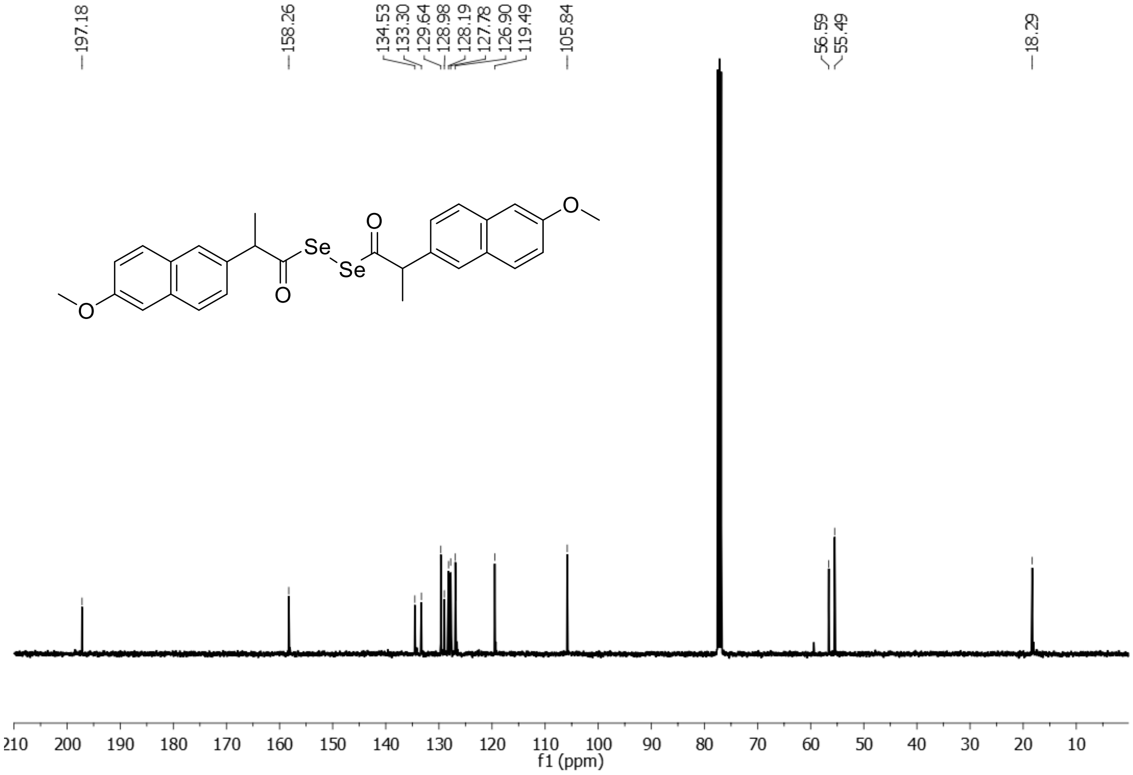

Figure S10. ^{13}C-NMR spectrum of compound **3**.

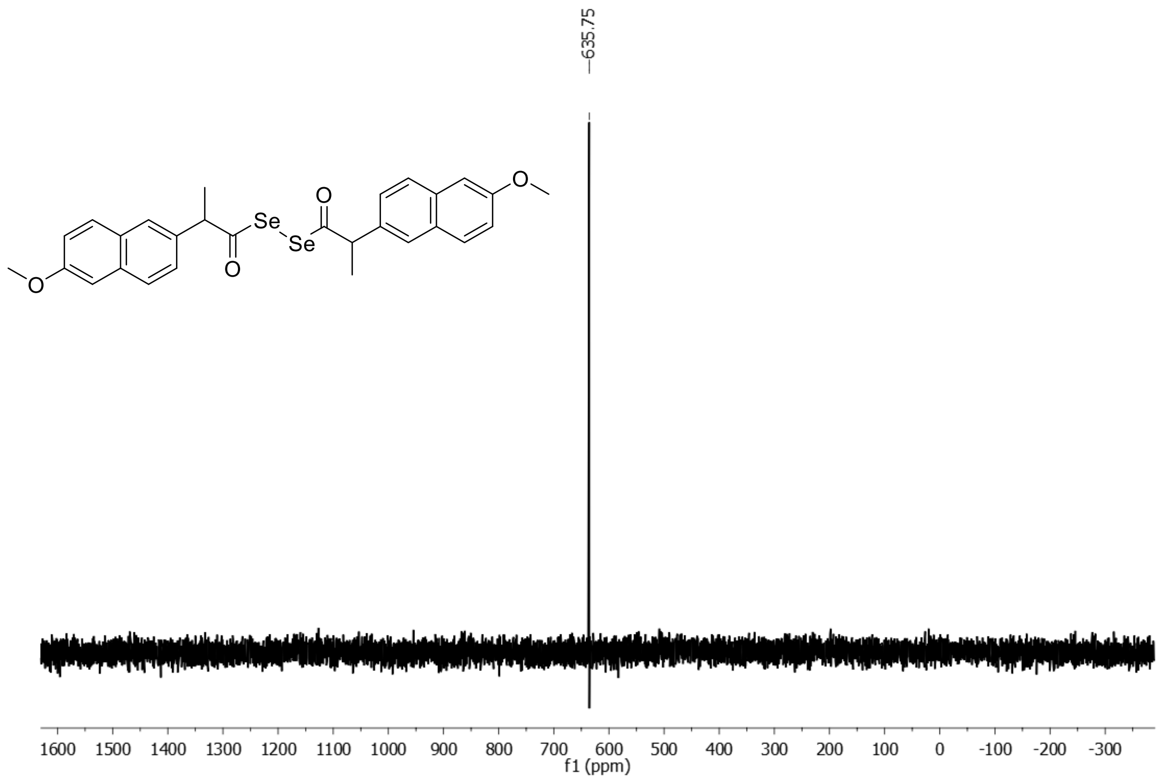

Figure S11. ^{77}Se-NMR spectrum of compound **3**.

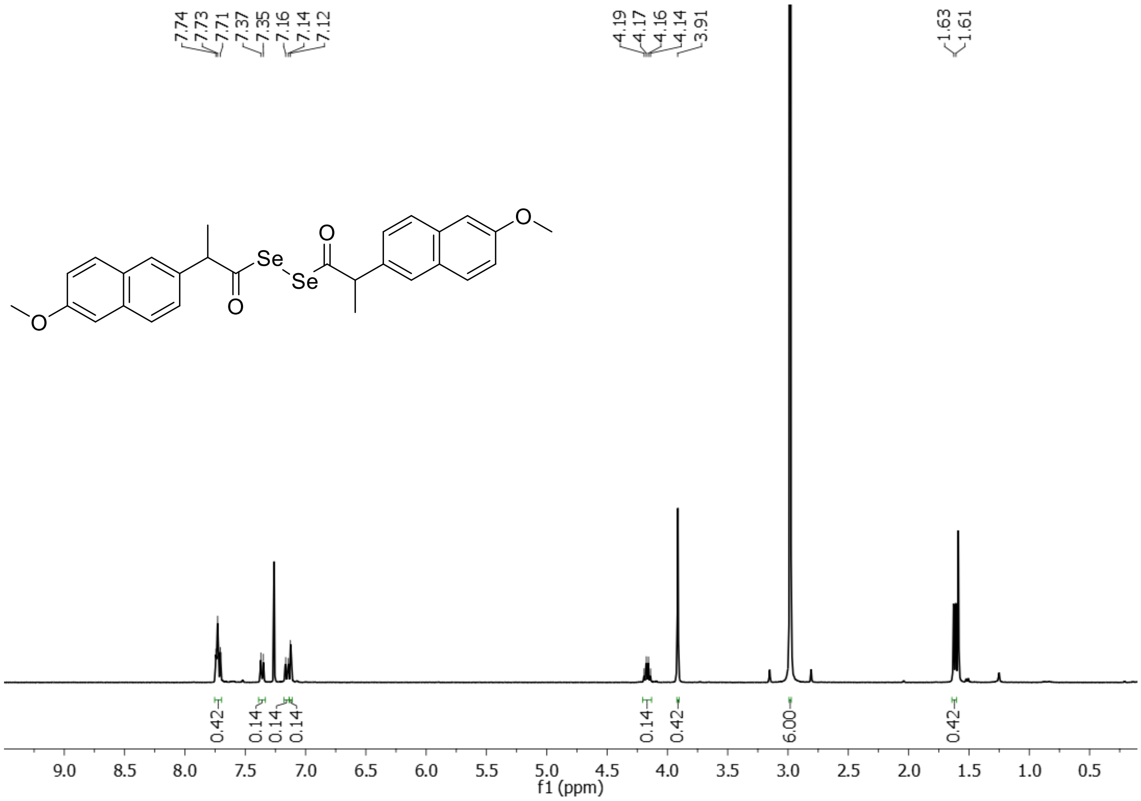

Figure S12. qNMR spectrum of compound **3**.

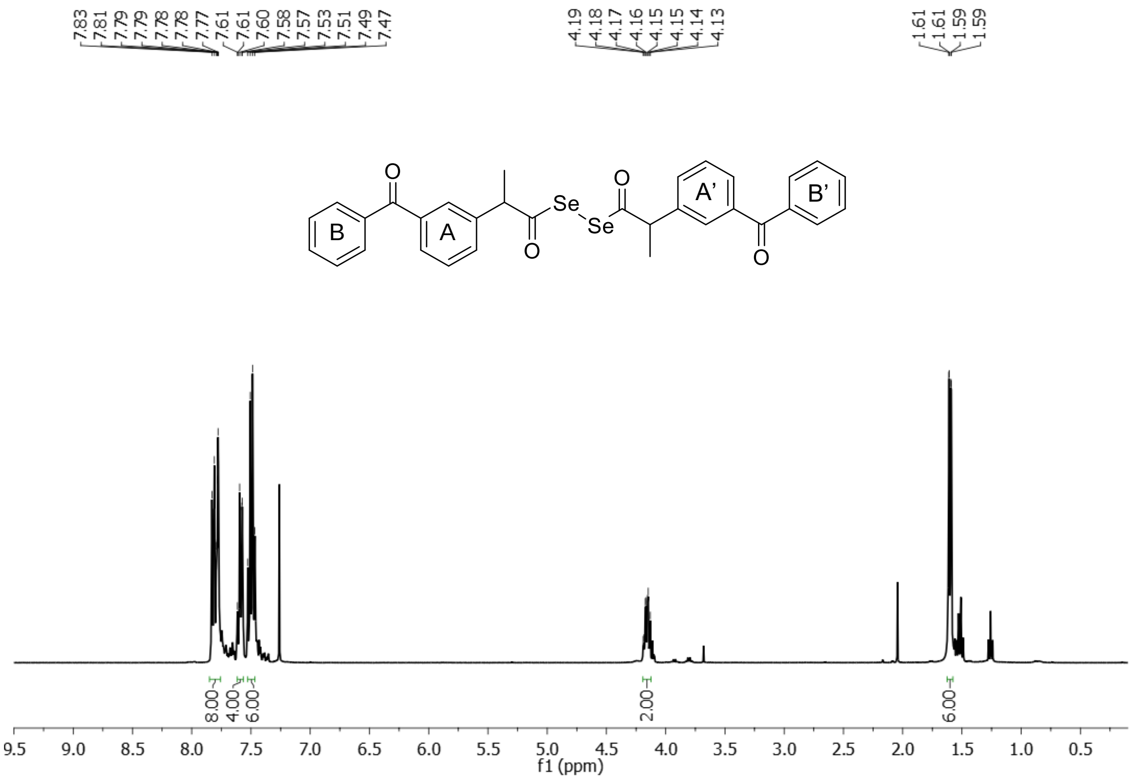

Figure S13. ^{1}H-NMR spectrum of compound **4**.

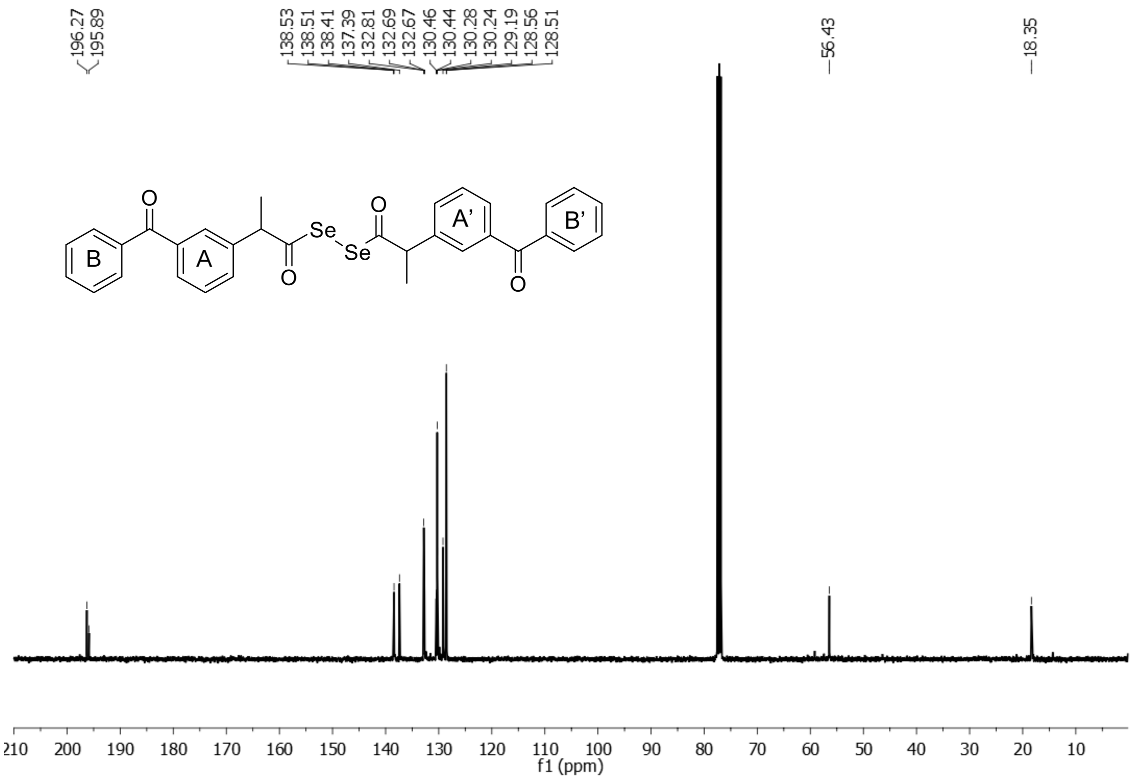

Figure S14. ^{13}C-NMR spectrum of compound **4**.

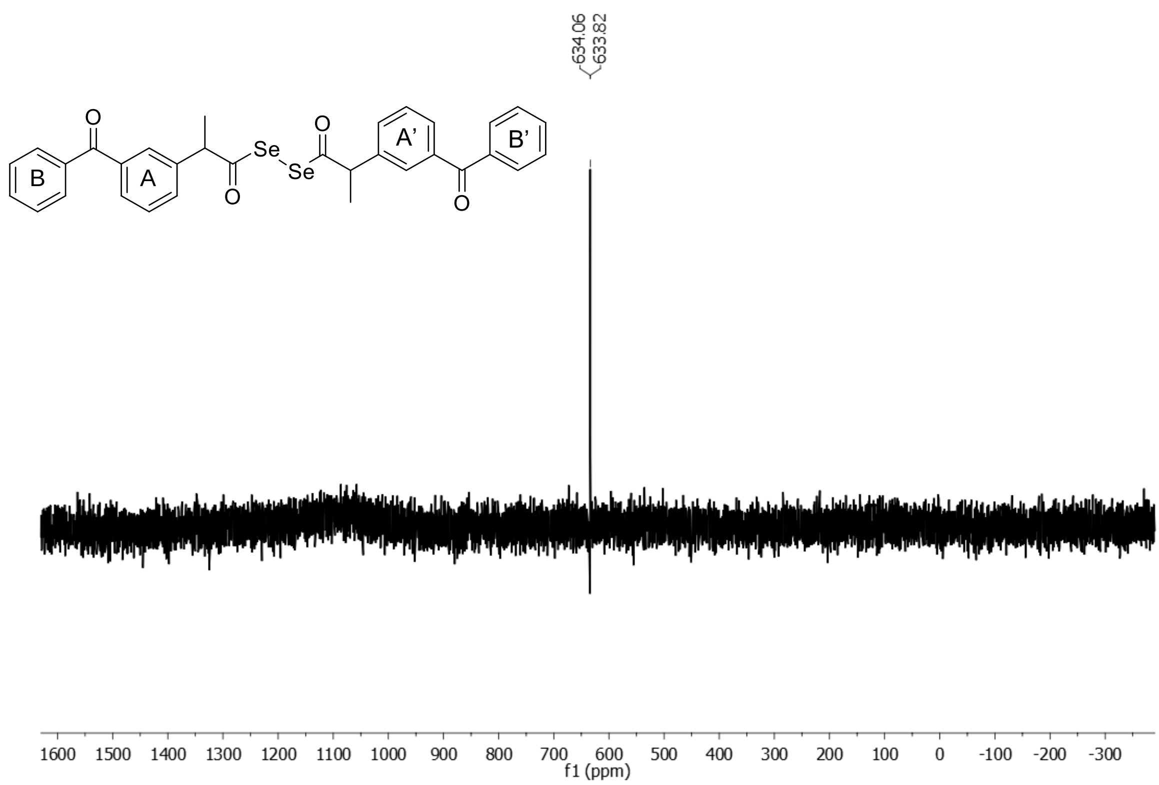

Figure S15. ^{77}Se-NMR spectrum of compound **4**.

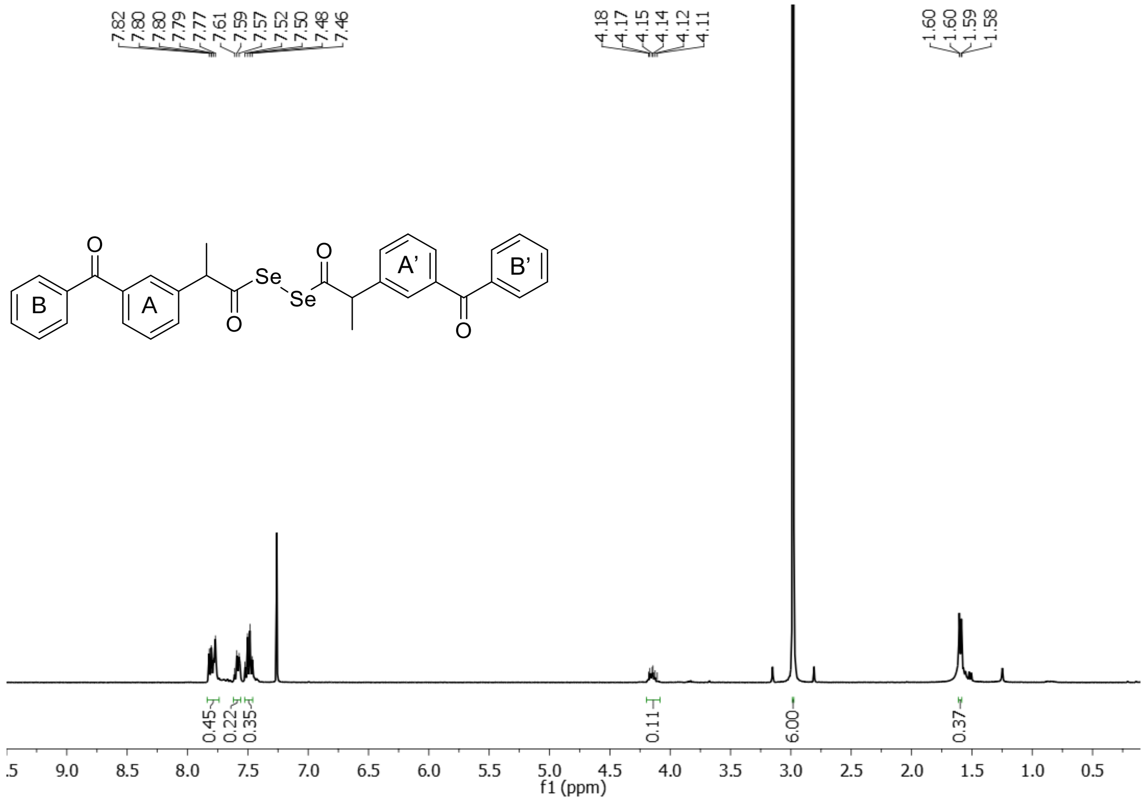

Figure S16. qNMR spectrum of compound **4**.

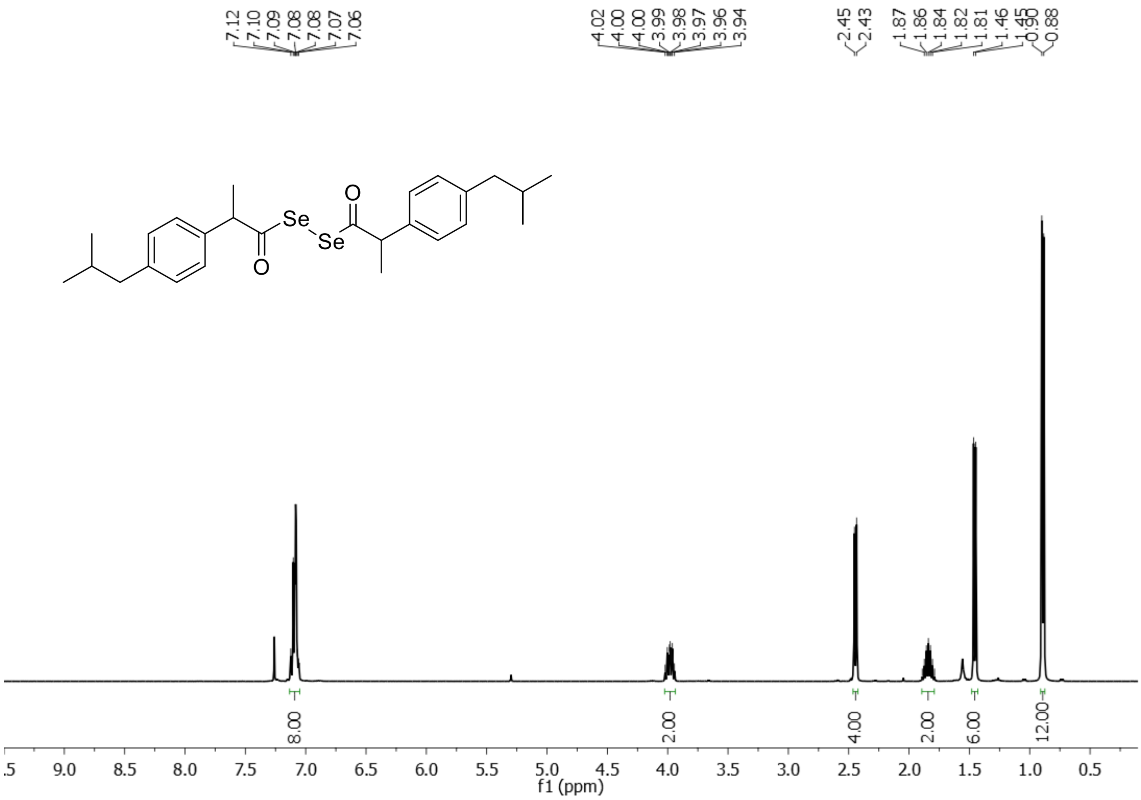

Figure S17. ^{1}H-NMR spectrum of compound **5**.

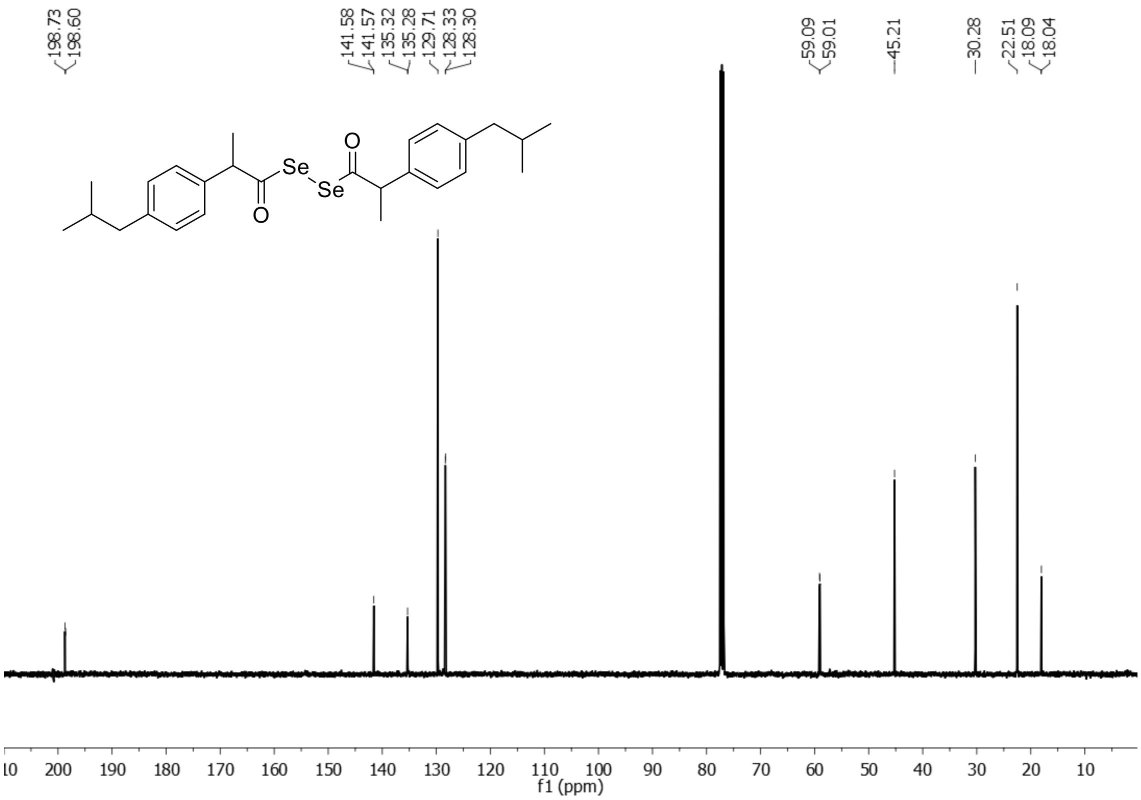

Figure S18. ^{13}C-NMR spectrum of compound **5**.

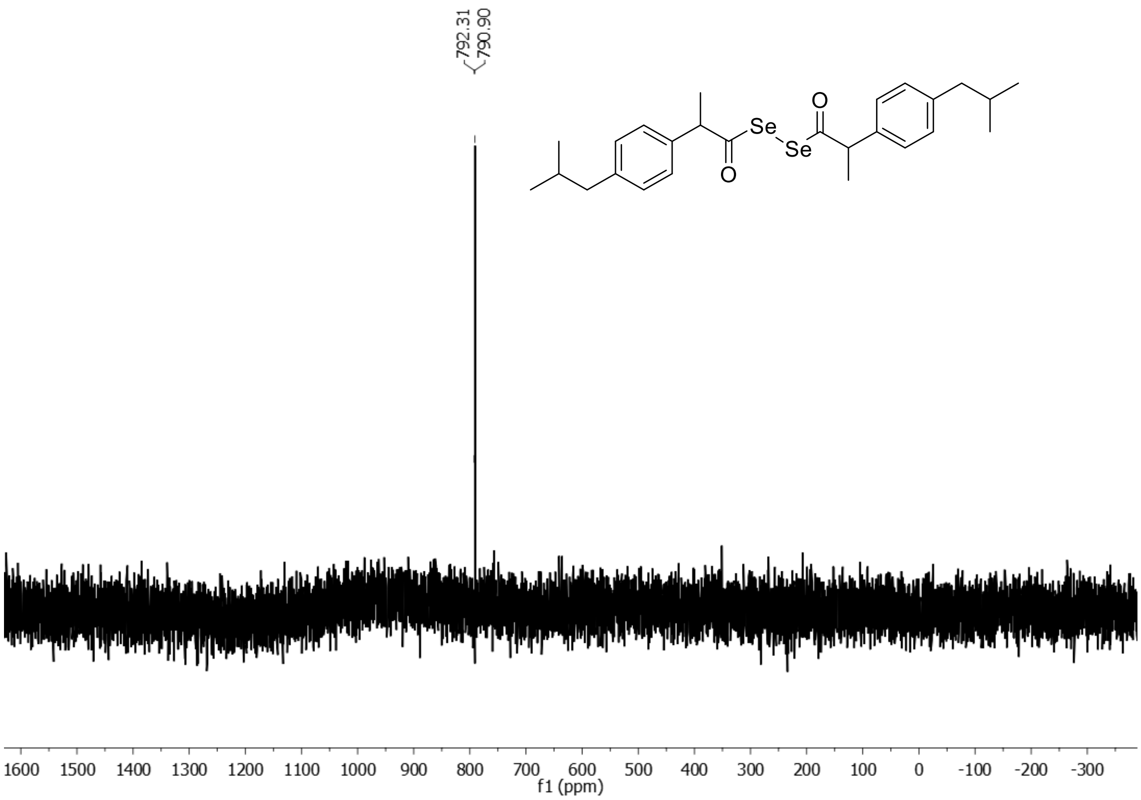

Figure S19. ^{77}Se-NMR spectrum of compound **5**.

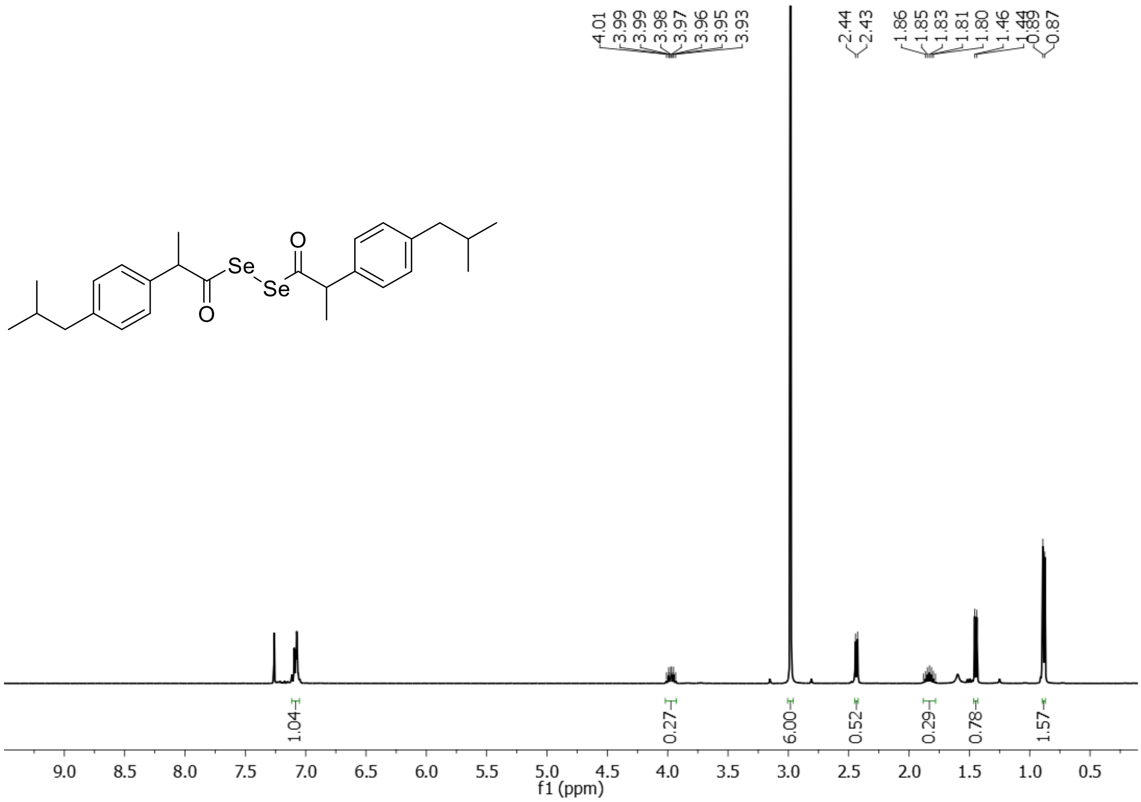

Figure S20. qNMR spectrum of compound **5**.

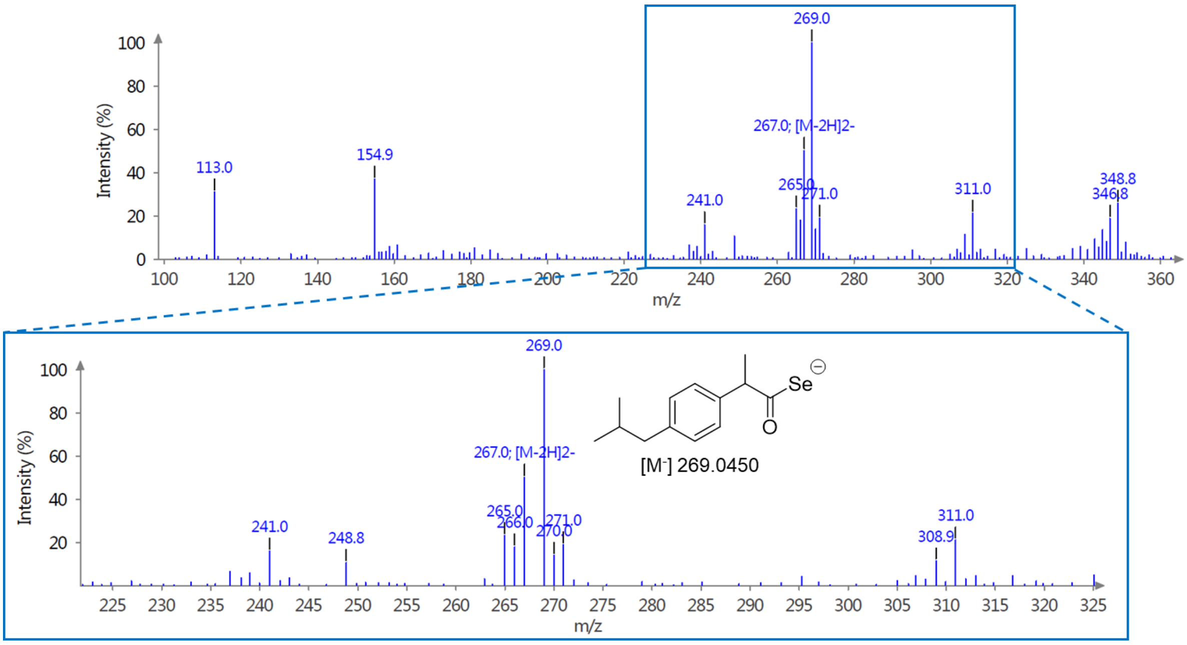

Figure S21. Mass spectrum of compound **5** obtained with a negative ionization mode.
